# Genome-Wide Identification and Characterization of CDPK Family Reveal Their Involvements in Growth and Development and Abiotic Stress in Sweet Potato and Its Two Diploid Relatives

**DOI:** 10.3390/ijms23063088

**Published:** 2022-03-13

**Authors:** Xu Li, Limeng Zhao, Huan Zhang, Qingchang Liu, Hong Zhai, Ning Zhao, Shaopei Gao, Shaozhen He

**Affiliations:** 1 Key Laboratory of Sweet Potato Biology and Biotechnology, Ministry of Agriculture and Rural Affairs/Beijing Key Laboratory of Crop Genetic Improvement/Laboratory of Crop Heterosis & Utilization and Joint Laboratory for International Cooperation in Crop Molecular Breeding, Ministry of Education, College of Agronomy & Biotechnology, China Agricultural University, Beijing 100193, China; lixu0207@cau.edu.cn (X.L.); limeng_zhao@cau.edu.cn (L.Z.); zhanghuan1111@cau.edu.cn (H.Z.); liuqc@cau.edu.cn (Q.L.); zhaihong@cau.edu.cn (H.Z.); zhaoning2012@cau.edu.cn (N.Z.); spgao@cau.edu.cn (S.G.); 2 Sanya Institute of China Agricultural University, Hainan 572025, China

**Keywords:** *Ipomoea batatas*, *Ipomoea trifida*, *Ipomoea triloba*, *CDPK*, tissue specificity, hormone treatment, abiotic stress

## Abstract

Calcium-dependent protein kinase (CDPKs) is one of the calcium-sensing proteins in plants. They are likely to play important roles in growth and development and abiotic stress responses. However, these functions have not been explored in sweet potato. In this study, we identified 39 *CDPKs* in cultivated hexaploid sweet potato (*Ipomoea batatas*, 2n = 6x = 90), 35 *CDPKs* in diploid relative *Ipomoea trifida* (2n = 2x = 30), and 35 *CDPKs* in *Ipomoea triloba* (2n = 2x = 30) via genome structure analysis and phylogenetic characterization, respectively. The protein physiological property, chromosome localization, phylogenetic relationship, gene structure, promoter *cis*-acting regulatory elements, and protein interaction network were systematically investigated to explore the possible roles of homologous *CDPKs* in the growth and development and abiotic stress responses of sweet potato. The expression profiles of the identified *CDPKs* in different tissues and treatments revealed tissue specificity and various expression patterns in sweet potato and its two diploid relatives, supporting the difference in the evolutionary trajectories of hexaploid sweet potato. These results are a critical first step in understanding the functions of sweet potato *CDPK* genes and provide more candidate genes for improving yield and abiotic stress tolerance in cultivated sweet potato.

## 1. Introduction

Ca^2+^ is an important second messenger in plants, and signaling pathways mediated by Ca^2+^ have been shown to play an important role in plant growth and development in response to abiotic and biotic stresses [1,2]. When cells sense external changes, the Ca^2+^ concentration in the cytoplasm changes, resulting in a series of physiological and biochemical reactions to plant tolerance improvement. There are three families of calcium-sensing proteins in plants, including calmodulin (CaM) and calmodulin-like protein (CaML), calcium-dependent protein kinase (CDPK), and calcineurin B-like proteins (CBLs)/CBL-interacting proteins (CIPK) [3,4,5,6,7]. Among these sensors, CDPKs are Ser/Thr protein kinases, serving as special sensors as they can directly convert upstream Ca^2+^ signals into downstream protein phosphorylation events [8]. Genome-wide analysis led to the identification of *CDPK* genes in various plant species. There are 34 genes in *Arabidopsis thaliana* [3], 31 in rice (*Oryza sativa*) [9], 20 in wheat (*Triticum aestivum*) [10], 35 in maize (*Zea mays*) [11], 29 in poplar (*Populus trichocarpa*) [12], and 19 in grape (*Vitis* spp.) [13].

CDPKs (CPKs) are single peptide chains with four typical domains: variable region, catalytic region (kinase region), linker region (autoinhibitory region), and regulatory region (calmodulin-like region/CaM-LD) [3,14]. The variable region is poorly conserved and generally contains 20 to 200 amino acid residues that may be involved in substrate recognition. Most CDPKs contain a palmitoylation site (cysteine residue at position 4 or 5) and a myristoylation site (glycine residue at position 2) related to membrane localization at the N-terminus [15]. The catalytic region includes all 11 highly conserved subdomains of eukaryotic Ser/Thr protein kinases, including the conserved Lys residue located in the second subdomain, which may be the binding site for ATP [16,17]. The linker region is composed of the most conserved 31 amino acids rich in basic amino acids, which can bind to the catalytic region to inhibit kinase activity, and also has a binding site to the regulatory region [17,18] which generally contains 1 to 4 (mostly 4) EF chiral structures that bind to Ca^2+^ [19].

Recent studies have shown that *CDPK* genes are involved in plant growth and development along with abiotic and biotic stress through hormone signaling [20,21,22,23,24,25,26]. In *Medicago truncatula*, silencing *CDPK1* expression resulted in significantly reduced root hair and root cell lengths [27]. Overexpression of *BnaCPK2* induced ROS accumulation and cell death [28]. *AtCPK17* and *AtCPK34* transduced Ca^2+^ signals to increase the rate of pollen tube tip growth [29]. *PnCDPK1* was involved in *Pharbitis nil* flowering [30]. As a regulatory component, *CPK28* from *Arabidopsis thaliana* regulated stem elongation and vascular development [31]. *OsCPK7* was induced by cold and salt stress, and its overexpression increased cold, salt, and drought tolerance [32]. AtCPK32 interacted with ABF4 and its overexpression affected ABA sensitivity [33]. The expression of *OsCDPK13* was increased in leaf sheath segments under gibberellin treatment or cold stress, suggesting that *OsCDPK13* might be an important signaling component of rice seedlings in response to gibberellins under cold stress conditions [34]. However, little is known about *CDPKs* in sweet potato.

Sweet potato (*Ipomoea batatas* (L.) Lam.), as an important food and feed crop, as well as an industrial and energy raw material [35], ranks 8th in terms of world food production [36]. Sweet potato is hexaploid (2n = 6x = 90) [37], with complex genomes, hybrid incompatibility, lack of germplasm resources, susceptibility to diseases and insect pests, etc. It is of great significance to enhance the adaptability of sweet potato to saline-alkali land and improve the yield of sweet potato through molecular mechanisms. Since 2017, the genome of sweet potato has been sequenced, assembled, and released, including a hexaploid sweet potato Taizhong 6 [37] and two diploid species *Ipomoea trifida* NCNSP0306 (2n = 2x = 30) and *Ipomoea triloba* NCNSP0323 (2n = 2x = 30) (Figure S1) [38]. Using whole-genome sequence data in sweet potato to identify and analyze important gene families is feasible.

In this research, a total of 109 *CDPKs* (i.e., 39 in *I. batatas*, 35 in *I. trifida*, and 35 in *I. triloba*) were identified from the cultivated hexaploid sweet potato and its two diploid relatives. They were classified into five subgroups. We systematically investigated the protein physiological property, chromosome localization, phylogenetic relationship, conserved motifs, *cis*-elements of the promoter, and protein interaction network of *CDPKs* in sweet potato. Moreover, the tissue specificity and expression pattern analysis for hormone response and abiotic stress of *CDPKs* were analyzed by qRT-PCR and RNA-seq. The evolution, different functions on development, hormone crosstalk, and abiotic stress response were also discovered between sweet potato and its two diploid relatives.

## 2. Results

### 2.1. Genome-Wide Identification and Characteristic of CDPKs Family in Sweet Potato and Its Two Diploid Relatives

In order to identify all *CDPKs* in sweet potato and its two diploid relatives, two typical strategies (i.e., hmmersearch, SMART, and CD-search databases) were employed. A total of 39, 35, and 35 *CDPKs* were identified in *I. batatas, I. trifida*, and *I. triloba*, respectively (named after “*Ib*”, “*Itf*”, “*Itb*”). The physicochemical properties of *CDPKs* were analyzed using the sequences from *I. batatas* (Table 1). The CDS length of *IbCDPKs* varied from 1056 bp (*IbCDPK25.4*) to 3513 bp (*IbCDPK16*). The amino acid lengths of IbCDPKs were distributed from 351 aa (IbCDPK25.4) to 1170 aa (IbCDPK16), the molecular weight (MW) ranged from 38.732 kDa to 130.824 kDa, and the isoelectric point (pI) varied from 4.75 (IbCDPK25.4) to 9.28 (IbCDPK28). Most IbCDPKs contained 4 EF-hands, except IbCDPK12.3, IbCDPK18, IbCDPK24, IbCDPK25.4, IbCDPK29.3, IbCDPK32, IbCDPK35. Ten IbCDPKs have no myristoylation sites in N-terminus (i.e., IbCDPK 1, IbCDPK11.2, IbCDPK11.3, IbCDPK12.1, IbCDPK12.2, IbCDPK12.3, IbCDPK24, IbCDPK25.1, IbCDPK29.1, IbCDPK35) and eight IbCDPKs have no palmitoylation sites (i.e., IbCDPK1, IbCDPK11.1, IbCDPK12.1, IbCDPK12.3, IbCDPK24, IbCDPK25.1, IbCDPK29.1, IbCDPK33.1).

**Figure 1:**
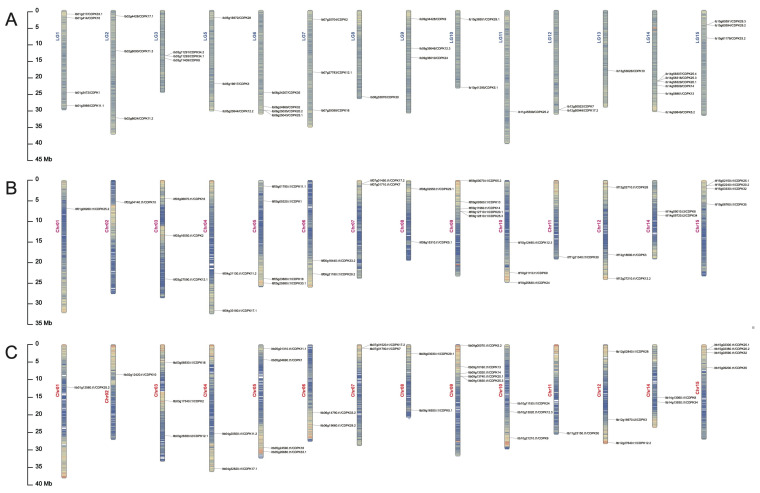
Chromosomal localization and distribution of *CDPK* genes in *I. batatas* (**A**), *I. trifida* (**B**), and *I. triloba* (**C**). The bars on the left margin represent chromosomes. The chromosome numbers are displayed on the left side of the chromosomes, and the gene names are displayed on the right side. Detail chromosomal location information is listed in Table S1.

All the *CDPKs* were separately mapped on 15 chromosomes of *I. batatas*, *I. trifida*, and *I. triloba* (Figure 1). In the *I. batatas* genome, 39 *IbCDPKs* genes were distributed across every chromosome except LG4. In the *I. trifida* and *I. triloba* genome, 35 *IbCDPKs* genes were distributed across every chromosome except Chr13. In *I. batatas*, four *IbCDPKs* were detected on LG1, three on LG2, three on LG3, three on LG5, four on LG6, three on LG7, one on LG8, three on LG9, two on LG10, one on LG11, two on LG12, one on LG13, six on LG14, and three on LG15 (Figure 1A). The numbers of *Itf/ItbCDPKs* located on the chromosomes were the same in two diploid relatives. One *CDPK* was detected on Chr01, Chr02, and Chr11; two on Chr04, Chr06, Chr07, Chr08, and Chr14; three on Chr03, Chr10, and Chr12; four on Chr05 and Chr15; and five on Chr09 (Figure 1B,C). The results indicated that the distribution of *CDPKs* was different on chromosomes in sweet potato and its two diploid relatives, whereas it was similar in two diploid relatives.

### 2.2. Phylogenetic Relationship of CDPKs in Sweet Potato and Its Two Diploid Relatives

**Figure 2:**
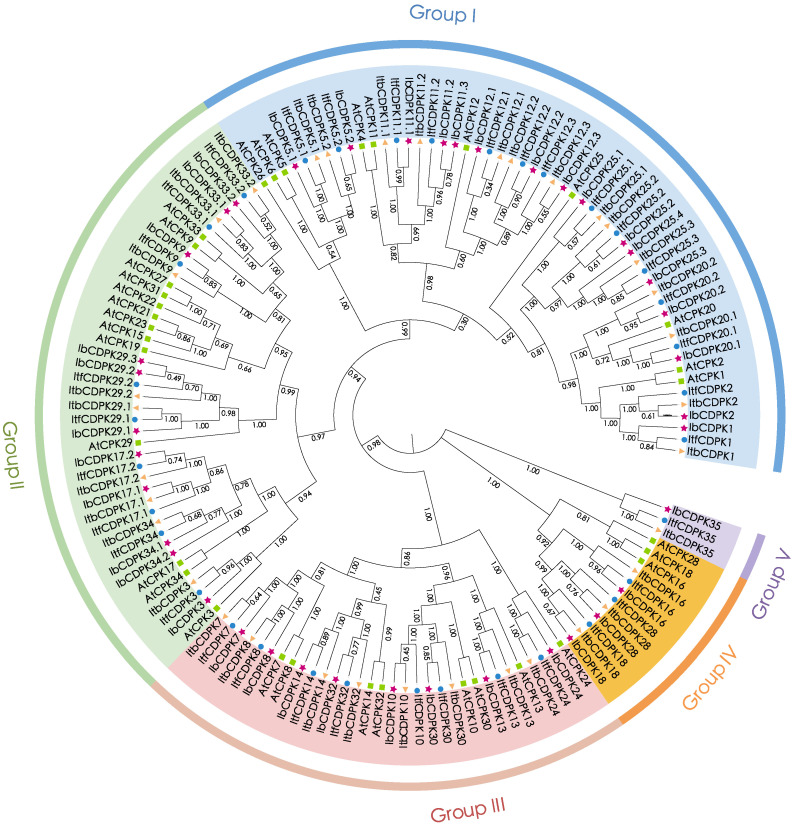
Phylogenetic analysis of the CDPK family in *I. batatas*, *I. trifida*, *I. triloba*, and *Arabidopsis*. A total of 143 CDPK*s* were divided into five subgroups (group I to V) according to the evolutionary distance. The pink pentagrams, blue cycles, yellow triangles, and green squares represent IbCDPKs in *I. batatas*, ItfCDPKs in *I. trifida*, ItbCDPKs in *I. triloba*, and AtCPKs in *Arabidopsis,* respectively.

To study the evolutionary relationship of CDPKs in *I. batatas*, *I. trifada*, *I. triloba*, and *Arabidopsis*, we constructed a phylogenetic tree for 143 CDPKs (i.e., 39 in *I. batatas*, 35 in *I. trifida*, 35 in *I. triloba*, and 34 in *Arabidopsis*) (Figure 2). All CDPKs were unevenly distributed and divided into five subgroups (group I to V) according to the evolutionary distance. The specific distribution of CDPKs was as follows (total: *I. batatas*, *I. trifida*, *I. triloba*, *Arabidopsis*): group I (54:16, 14, 14, 10); group II (42:11, 9, 9, 13); group III (32:8, 8, 8, 8); group IV (12:3, 3, 3, 3), and group V (3:1, 1, 1, 0) (Figure 2; Table S1). We named IbCDPKs, ItfCDPKs, and ItbCDPKs based on their homology with homologs in *Arabidopsis*, and AtCDPK15/19/21/22/23/27/31 from *Arabidopsis* have no homologous protein in *I. batatas*, *I. trifada*, *I. triloba*, and only Ib/Itf/ItbCDPK35 have no homologous proteins in *Arabidopsis*. One additional IbCDPK sequence (IbCDPK25.4) was identified in *I. batatas* that had no homology protein in *I. trifida* and *I. triloba.* Two sequences (IbCDPK34.1 and IbCDPK34.2) in *I. batatas* were homologous with ItfCDPK34 and ItbCDPK34. We speculated that large differences in number and type of CDPKs divided in five subgroups between *Arabidopsis* and sweet potato and its two diploid relatives were due to species specificity. Moreover, the discrepancy showed in sweet potato and its two diploid relatives might be due to chromosomal hybridization during evolution.

### 2.3. Conserved Motif and Exon-Intron Structure Analysis of CDPKs in Sweet Potato and Its Two Diploid Relatives

**Figure 3:**
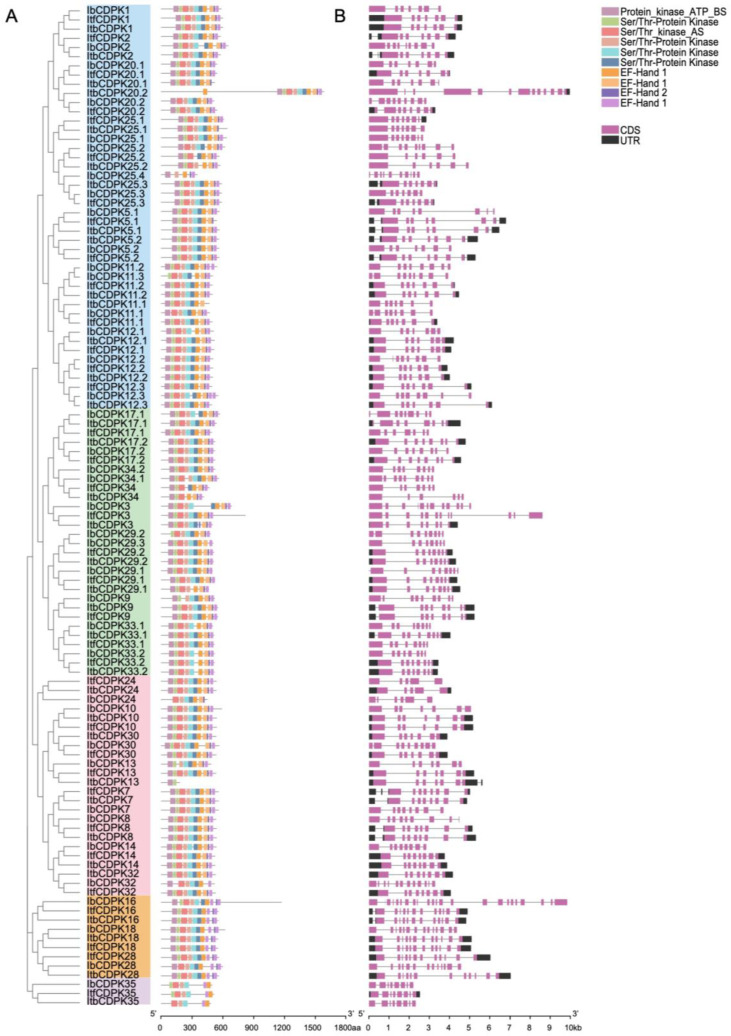
Conserved motifs and exon–intro structure analysis of *IbCDPKs, ItfCDPKs*, and *ItbCDPKs* family. (**A**) Ten conserved motifs of CDPKs are shown in different colors. (**B**) Exon–intron structures of *IbCDPKs, ItfCDPKs*, and *ItbCDPKs*. The purple boxes, black boxes, and black lines represent the CDS, UTRs, and introns, respectively.

To illustrate the structural characteristics of the 109 CDPKs from *I. batatas*, *I. trifida*, and *I. triloba*, we performed motif and domain analysis using the MEME website (Figure 3). A total of 10 motifs were identified (Figure 3A and Figure S2). Overall, the protein structure of this family was relatively conserved, and most of the CDPKs contained six protein kinase domains and four EF-hands (three EF-hand_1 and one EF-hand_2) except ItbCDPK13. Most CDPKs contained even numbers of EF-hand except IbCDPK11.2, -12.3, -24, -25.3, -29.3, ItfCDPK5.1, and ItbCDPK20.1, -20.2, -25.1. The composition of the EF-hands of Ib/Itf/ItbCDPKs in group V was different, with two EF-hand_1 and no EF-hand_2. The number of protein kinase domains and EF-hands contained in individual protein varied in sweet potato and its two diploid relatives. IbCDPK11.3, -12.1, -20.2, -25.4 in group I, -9, ItbCDPK29.1, IbCDPK29.2, -29.3, -33.1 in group II, IbCDPK13 in group III, and IbCDPK28 in group IV lacked at least one protein kinase domain. In group I, ItfCDPK5.1, ItbCDPK20.1, and ItbCDPK25.1 contained two EF-hand_1 and one EF-hand_2. The C-terminus of IbCDPK12.3 and the N-terminus of ItbCDPK20.2 contained one more EF-hand. Ib/ItbCDPK11.1 lacked one and two protein kinase domains, respectively. Ib/Itf/ItbCDPK25.2 lacked one, two, and two protein kinase domains, respectively. In group II, the composition of the EF-hand of ItbCDPK3 was distinct, with two EF-hand_1 and two EF-hand_2. IbCDPK24 in group III lacked two protein kinase domains and one EF-hand_1. IbCDPK32 lacked one protein kinase domain and two EF-hands. Itf/ItbCDPK34 lacked two and three protein kinase domains, respectively. In group V, Ib/ItfCDPK35 lacked two protein kinase domains and two EF-hands, and ItbCDPK35 lacked three protein kinase domains and two EF-hands.

The exon–intron structures of *IbCDPKs* varied from those of *Itf/ItbCDPKs*, with the coding DNA sequence (CDS) composition ranging from five to twenty-three exons (Figure 3B). *Ib/Itf/ItbCDPKs* contained seven to ten, six to eight, and six to thirteen exons in group I; eight to twelve, seven to nine, and six to eight exons in group II; six to twelve, five to eight, and five to eight exons in group III; thirteen to twenty-three, twelve, and twelve exons in group IV; eight, eight, and eight exons in group V, respectively (Figure 3B).

The number of exon–intron structures was different in both sweet potato and its two diploid relatives and varied from *I. trifida* and *I. triloba* also. Some *CDPKs* contained same exon-intron in *I. trifida* and *I. triloba* but less than those in *I. batata* (i.e., *Ib/Itf/ItbCDPK2*, *-CDPK5.1*, *-CDPK11.1*, *-CDPK11.2*, *-CDPK12.2*, *-CDPK12.3*, *-CDPK25.1*, *-CDPK25.2* in group I, *-CDPK9*, *-CDPK29.2* in group II; *-CDPK8*, *-CDPK24*, *-CDPK30*, *-CDPK32* in group III; all *CDPKs* in group IV) (Figure 3B). In group I, *ItfCDPK20.2* contained eight exons and its homologous gene (*ItbCDPK20.2*) in *I. triloba* contained thirteen exons, whereas *IbCDPK20.2* contained nine exons. In group II, *IbCDPK3* contained twelve exons, *ItbCDPK3* contained nine exons, whereas *IbCDPK3* contained eight exons. *IbCDPK17.1* contained nine exons, *ItbCDPK17.1* contained seven exons, whereas *ItfCDPK17.1* contained eight exons (Figure 3B).

### 2.4. Cis-Element Analysis in the Promoter of IbCDPKs in Sweet Potato

**Figure 4:**
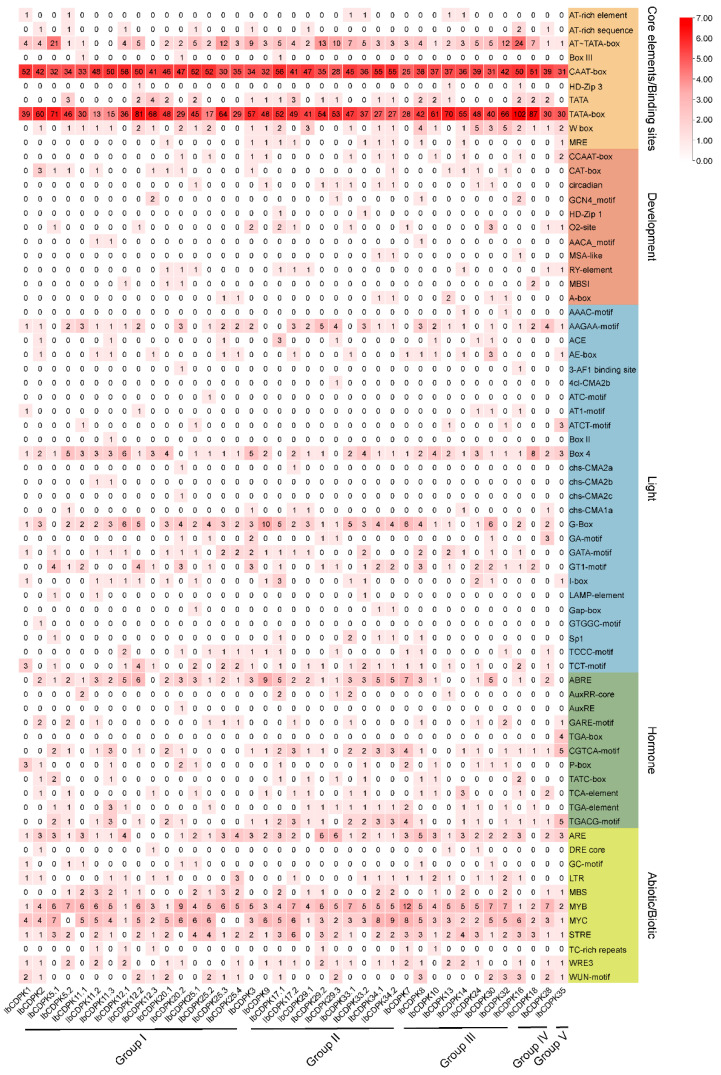
*Cis*-elements analysis of *IbCDPKs*. The *cis*-elements were divided into five groups. The degree of red colors represents the number of *cis*-elements upstream of the *IbCDPKs*.

*Cis*-acting elements are nucleotide sequences that are found upstream or downstream of genes and can regulate their transcription levels. They work through combining with some specific transcription factors when plants respond to various development processes and stresses. To reveal how *CDPKs* function in growth and development and abiotic stress adaption in sweet potato, 2000 bp upstream sequences of 39 *IbCDPKs* in *I. batatas* were extracted and the *cis*-element analysis was performed using PlantCARE website (http://bioinformatics.psb.ugent.be/webtools/plantcare/html/, accessed on 30 January 2022). According to the prediction function, all *cis*-elements were divided into core elements and binding sites, development, light-responsive, hormonal-responsive, and abiotic/biotic stress-responsive elements (Figure 4). The degree of red colors represented the number of *cis*-elements upstream of the *IbCDPKs*.

**Figure 5:**
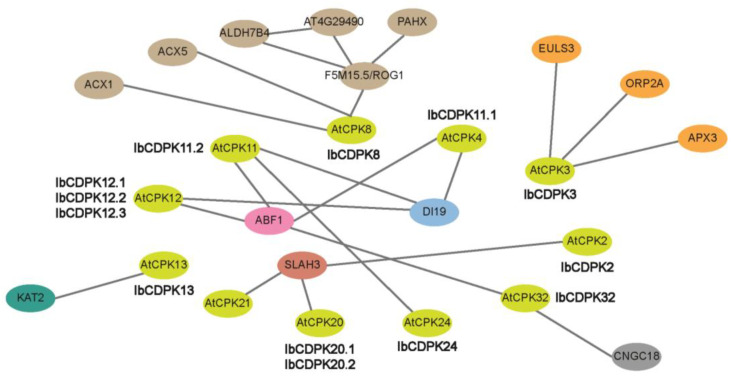
Protein interaction network of IbCDPKs in *I. batatas* according to orthologues in *Arabidopsis*. Network nodes represent proteins, green nodes represent AtCPKs and other colored nodes represent interacting proteins. Lines represent protein–protein interaction which was experimentally determined.

The majority of 39 *CDPKs* possessed a large number of core promoter elements and binding sites, such as AT-TATA-box, TATA-box, and CAAT-box (Figure 5). TATA-box and CAAT-box are the binding sites of RNA polymerase and are involved in the transcription initiation and frequency of genes [39]. Some development elements (i.e., CCAAT-box, CAT-box, circadian, O2-site, RY-element, and A-box) were found in *IbCDPKs* (Figure 4). Light-responsive elements (i.e., AAGAA-motif, Box 4, G-box, CATA-motif, GT1-motif, Sp1, TCCC-motif, and TCT-motif) were found in most of *IbCDPKs* (Figure 4). In addition, hormonal-responsive elements (i.e., ABA-responsive element ABRE, GARE-motif, MeJA-responsive elements CGTCA-motif and TGACG-motif, GA-responsive elements P-box and TATC-box, SA-responsive element TCA, auxin-responsive element TGA-element) were found. The majority of *IbCDPKs* except *IbCDPK1*, *IbCDPK12.3*, and *IbCDPK13*, processed at least two hormone-responsive elements (Figure 4). These results indicated that *IbCDPKs* might be involved in the crosstalk between different hormone signaling pathways. Furthermore, anaerobic induction-responsive element ARE, low temperature-responsive element LTR, drought-responsive elements MBS, MYB and MYC, stress-responsive element STRE, injury and defensive-responsive elements WRE3, and WUN-motif were found in most *IbCDPKs* (Figure 4). All *IbCDPKs* processed at least three drought-responsive elements. These results suggested that *IbCDPKs* might be involved in the crosstalk between hormone signaling pathways to regulate the growth and development and stress adaption in sweet potato, particularly in drought stress.

### 2.5. Protein Interaction Network of IbCDPKs

To explore the potential regulatory network of IbCDPKs, we constructed an IbCDPKs interaction network based on *Arabidopsis* orthologous proteins (Figure 5). We speculated IbCDPK11 might interact with IbCDPK24. They also interacted with JA biosynthesis-related protein (i.e., ACX1 and ACX5), ABA-responsive element-binding factor 1 (ABF1), potassium channel protein (KAT2), and stomatal movement protein (ELUS3). They could interact with L-ascorbate peroxidase 3 (APX3) and catalase (F5M15.5/ROG1) to scavenge hydrogen peroxide in plants. IbCDPK11.1, IbCDPK11.2, IbCDPK12.1, IbCDPK12.2, and IbCDPK12.3 might interact with DI19 (DEHYDRATION-INDUCED 19) in response to drought stress. IbCDPK2 might generate a complex with IbCDPK20.1/20.2 through SALH3. SALH3, encoding S-type anion channel protein, was an essential negative regulator of inward potassium channels in guard cells. IbCDPK2, IbCDPK3, IbCDPK13, and IbCDPK20.1/20.2 may be essential for efficient stomatal movement in guard cells. IbCDPK3 might also interact with ORP2A to be involved in the transport of sterols. IbCDPK32 could interact with CNGC18 to regulate pollen growth in sweet potato. IbCDPK3 and IbCDPK8 might play important roles in disease resistance through their involvement in stomatal movement and JA biosynthesis. These results indicated that IbCDPKs might play an important role in plant growth and development, such as stomatal movement, icon transport, and participate in hormone signaling pathways (i.e., JA and ABA) in response to abiotic and biotic stresses. Therefore, interacting proteins of IbCDPKs are still worth exploring.

### 2.6. Expression Analysis of CDPKs in Sweet Potato and Its Two Diploid Relatives

#### 2.6.1. Expression Analysis in Various Tissues

**Figure 6:**
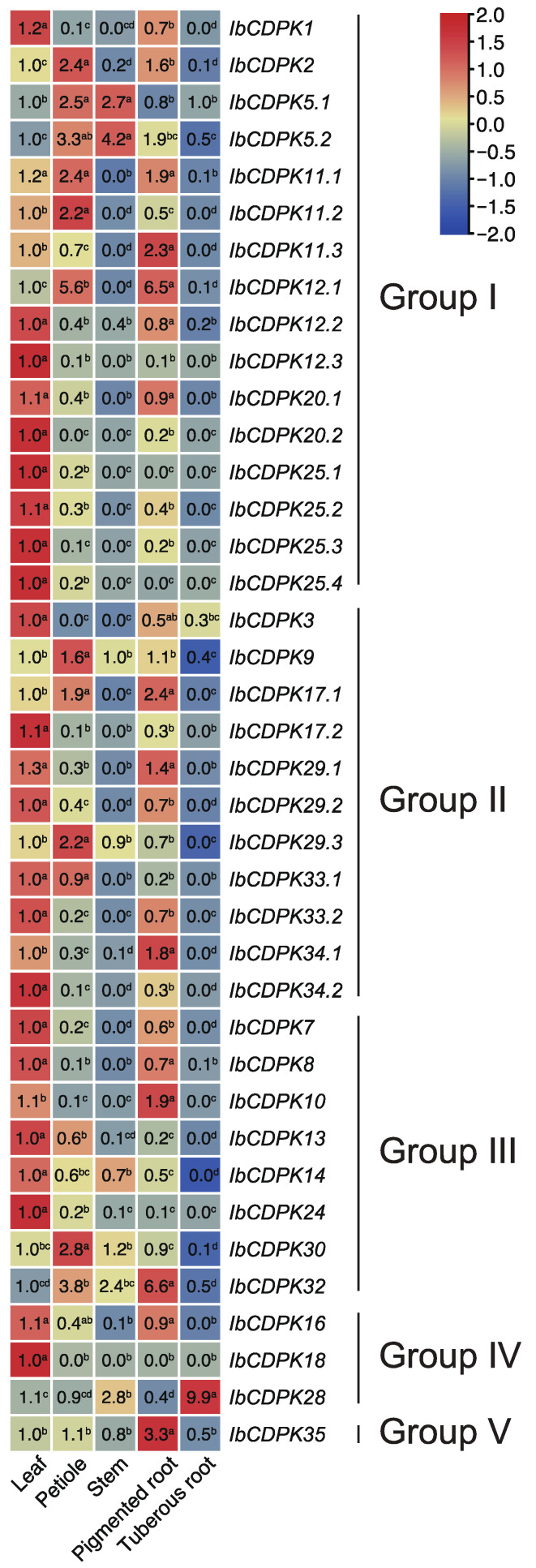
Gene expression patterns of *CDPKs* in leaf, petiole, stem, pigmented root, and tuberous root of *I. batatas.* The values were determined by RT-qPCR from three biological replicates consisting of pools of three plants, and the results were analyzed using the comparative C_T_ method. The fold change is shown in the boxes. Different lowercase letters indicate significant differences (*p* < 0.05; Student’s *t*-test).

To investigate the potential biological functions of *IbCDPKs* in growth and development, the expression level in five representative tissues (i.e., leaf, petiole, stem, pigmented root, and tuberous root) of *I. batatas* was analyzed using real-time quantitative PCR (qRT-PCR) (Figure 6). In general, different subgroups did not exhibit regular expression patterns in five tissues. Some *IbCDPKs* showed tissue-specific expression. Almost half of the *IbCDPKs* (i.e., *IbCDPK1, -12.3, -20.2, -25.1, -25.2, -25.3, -25.4, -3, -17.2, -29.2, -33.2, -34.2, -7, -13, -14, -24*, and *-18*) were preferably expressed in the leaf. Five *IbCDPKs* (i.e., *IbCDPK2,* *11.2*, *-9*, *-29.3*, and *-30*) were highly expressed in the petiole. *IbCDPK5.2* was highly expressed in the stem. Six *IbCDPKs* (i.e., *IbCDPK11.3*, *-12.1*, *-34.1*, *-10*, *-32*, and *-35*) showed a higher gene expression in pigmented root as compared with other tissues. Two *IbCDPKs* (i.e., *IbCDPK12.3*, *IbCDPK18*) showed low expression in all tissues. The majority of *IbCDPKs* showed a relatively higher gene expression in at least two tissues. *IbCDPK5.1* showed a higher expression level in the petiole and stem. *IbCDPK12.2, -20.1, -29.1, -8*, and *-16* showed a higher expression level in the leaf and pigmented root. *IbCDPK5.1* showed a higher expression level in the petiole and stem. *IbCDPK11.1* showed a higher expression level in the leaf, petiole, and pigmented root. *IbCDPK12.2*, -*20.1*, *-29.1*, -*8*, and -*16* showed a higher expression level in the leaf and pigmented root. *IbCDPK33.1* showed a higher expression level in the leaf and petiole. *IbCDPK28* showed a higher expression level in the tuberous root. These results indicated that *IbCDPKs* might function differently in different tissues of sweet potato.

**Figure 7:**
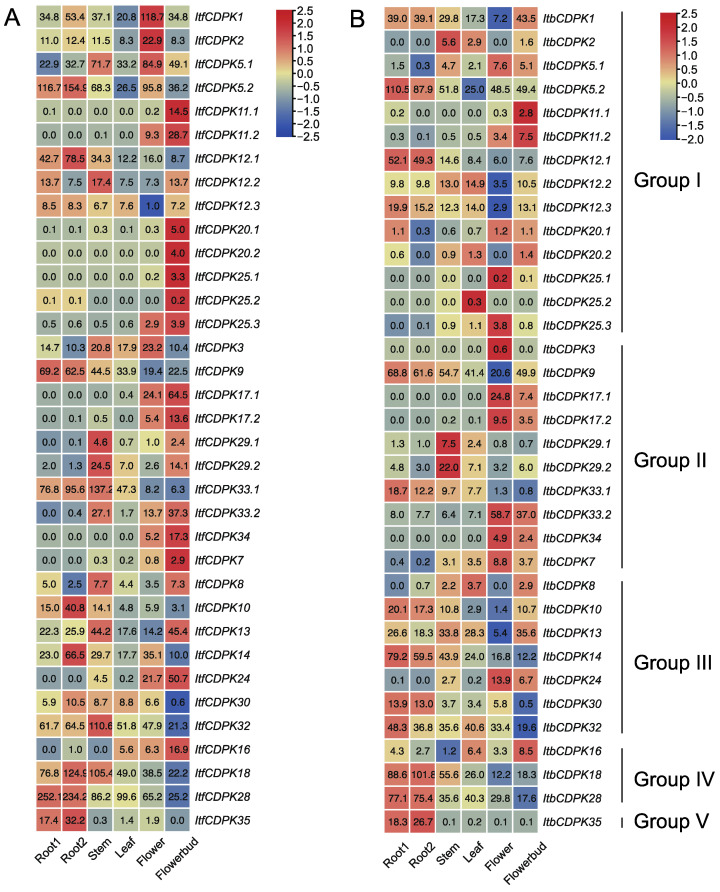
Gene expression patterns of *ItfCDPKs* (**A**) and *ItbCDPKs* (**B**) in root 1, root 2, stem, leaf, flower and flower bud of *I. trifida* as determined by RNA-seq. Log_2_ (FPKM + 1) is shown in the boxes.

Variation in gene expression between homolog *CDPKs* was also observed. RNA-seq data of six tissues (i.e., flower, flower bud, leaf, root 1, root 2, and stem) were used to analyze the expression patterns of *ItfCDPKs* and *ItbCDPKs* in *I. trifida* and *I. triloba,* respectively [38] (Figure 7). In *I. trifida*, eight *ItfCDPKs* (i.e., *ItfCDPK5.2*, *-12.1*, *-9*, *-10*, *-14, -18, -28*, and *-35*) were highly expressed in the root (root1 and root2). Five *ItfCDPKs* (i.e., *ItfCDPK12.2*, *-29.1*, *-29.2*, *-33.1*, and *-32*) were highly expressed in the stem. No *ItfCDPKs* were highly expressed in the leaf. Only two *ItfCDPKs* (i.e., *ItfCDPK1* and *ItfCDPK2*) were highly expressed in the flower. Fourteen of thirty-five *ItfCDPKs* (i.e., *ItfCDPK11.1*, *-11.2*, *-20.1*, *-20.2*, *-25.1, -25.2, -25.3, -17.1, -17.2, -33.2, -34, -7, -24*, and *-16*) were highly expressed in flowerbud. Furthermore, *ItfCDPK12.3, -8*, and *-30* showed low expression in all tissues, and *ItfCDPK12.3* showed the lowest expression in the flower, while *ItfCDPK30* had the lowest expression in *the* flower bud. *ItfCDPK5.1* and *ItfCDPK3* were highly expressed in the stem and flower, while *ItfCDPK13* was highly expressed in the stem and flowerbud (Figure 7A).

In *I. triloba*, the expression pattern *of ItbCDPKs* was similar to that *of ItfCDPKs* in *I. trifida* except for individual genes. *ItbCDPK1* showed low expression in the flower but not the leaf. *ItbCDPK2* was highly expressed in the stem but not flower. *ItbCDPK12.2* and *ItbCDPK25.2* were highly expressed in the leaf (Figure 7B). These results suggested that *CDPKs* had similar expression patterns and they might play the same role in the growth and development of *I. trifida* and *I. triloba*.

#### 2.6.2. Expression Analysis of Hormone Response

**Figure 8:**
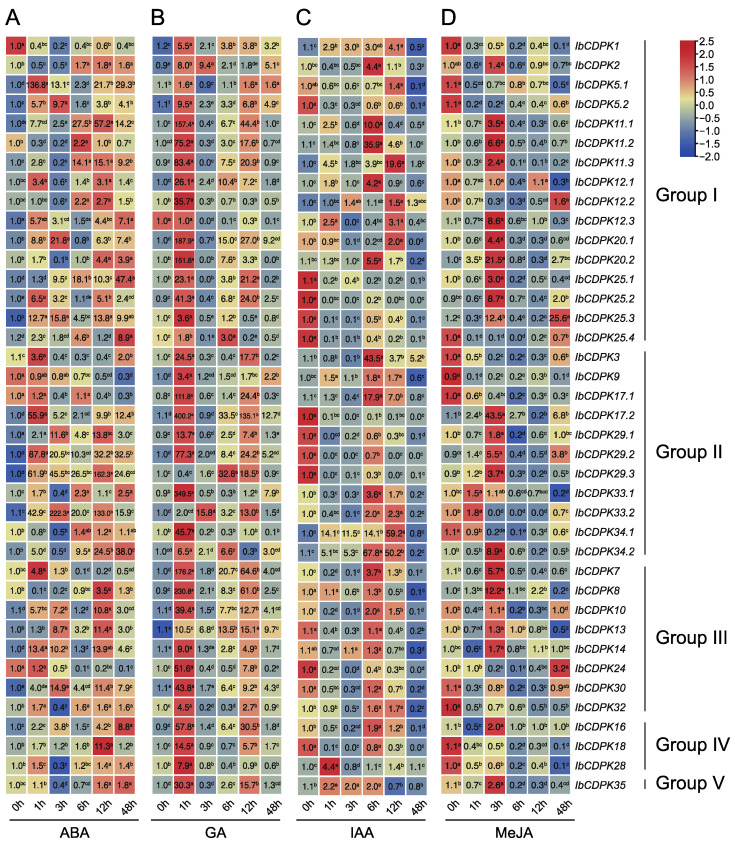
Gene expression patterns of *IbCDPKs* in response to different phytohormones, i.e., (**A**) ABA, (**B**) GA, (**C**) IAA, and (**D**) MeJA of *I. batatas.* The values were determined by RT-qPCR from three biological replicates consisting of pools of three plants, and the results were analyzed using the comparative C_T_ method. The expression of 0 h in each treatment was considered “1”. The fold change is shown in the boxes. Different lowercase letters indicate significant differences (*p* < 0.05; Student’s *t*-test).

Plant hormones regulate various processes of plant growth, development, and environmental adaptation. They both independently and cooperatively regulate plant seed germination [40,41,42], vegetative growth [43,44,45], reproductive growth [46], embryonic development [47], seed maturation [48,49], as well as the tolerance to biotic [50,51,52,53,54,55,56] and abiotic stresses [57,58]. Thus, we performed qRT-PCR to evaluate the expression level of *IbCDPKs* in response to hormones, including ABA, GA, IAA, and MeJA. Under ABA treatment, most *IbCDPKs* were induced, specifically *IbCDPK5.1*, *-29.3*, and *-33.2* which were up-regulated by 136.8-fold, 162.3-fold, and 222.3-fold, respectively. Two *IbCDPKs* (i.e., *IbCDPK1* and *IbCDPK9*) were repressed. Thirty *IbCDPKs* peaked within 12 h, while only seven *IbCDPKs* (i.e., *IbCDPK12.3*, *-25.1*, *-25.4*, *-33.1*, *-34.2*, *-16*, *-35*) peaked at 48 h (Figure 8A). Under GA treatment, almost all *IbCDPKs* were significantly induced at 1 h, with *IbCDPK17.2* and *IbCDPK33.1* showing the highest folds, by 400.2-fold and 349.5-fold, respectively, while *IbCDPK12.3* was repressed (Figure 8B). Under IAA treatment, twelve *IbCDPKs* (i.e., *IbCDPK5.2*, *-25.1*, *-25.2*, *-25.3*, *-25.4*, *-17.2*, *-29.1*, *-29.2*, *-29.3*, *-13*, *-24*, *-18)* were down-regulated. The expression levels of the most induced *IbCDPKs* (i.e., *IbCDPK2, -11.1, -11.2, -11.3, -12.1, -12.2, -12.3, -20.1, -20.2, -3, -17.1, -33.1, -34.1, -34.2, -7*) peaked at 6 h or 12 h. Only *IbCDPK28* peaked at 1 h (Figure 8C). Under MeJA treatment, twenty-five *IbCDPKs* were significantly up-regulated (two peaked at 1 h, twenty peaked at 3 h, and three peaked at 48 h), but twelve *IbCDPKs* (i.e., *IbCDPK1*, *-5.1*, *-5.2*, *-25.4*, *-3*, *-9*, *-17.1*, *-34.1*, *-30*, *-32*, *-18*, *-28*) were repressed (Figure 8D). All *IbCDPKs* were induced by at least two hormones. These results suggested that *IbCDPKs* showed different expression patterns in response to various hormone treatments and might participate in the crosstalk between multiple hormones.

**Figure 9:**
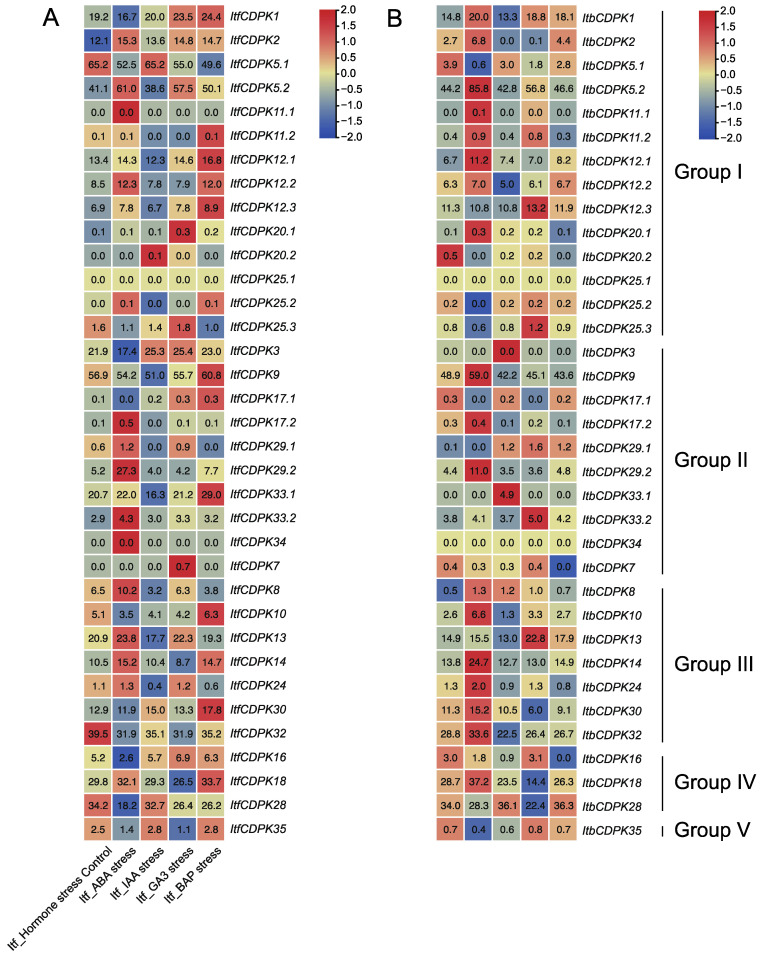
Gene expression patterns of *ItfCDPKs* (**A**) and *ItbCDPKs* (**B**) in response to different phytohormone (ABA, IAA, GA3, and BAP) in *I. trifida* and *I. triloba* as determined by RNA-seq. Log_2_ (FPKM + 1) is shown in the boxes.

We also analyzed the expression patterns of *ItfCDPKs* and *ItbCDPKs* using the RNA-seq data of *I. trifida* and *I. triloba* under ABA, IAA, GA, and BAP treatments [38]. In *I. trifida*, compared with the control (Itf_Control), *CDPKs* were induced by at least one hormone except for *ItfCDPK25.1*. Under ABA treatment, *ItfCDPK11.1*, *-17.1*, *-29.2*, *-33.2*, and *-34* were significantly induced while *ItfCDPK1*, *-3*, *-16*, and *-28* were repressed. Under IAA treatment, *ItfCDPK20.2* was up-regulated. *ItfCDPK5.2*, *-12.1*, *-12.3*, *-25.2*, *-9*, *-33.1*, *-13*, and *-24* were down-regulated. *ItfCDPK20.1*, *ItfCDPK25.3*, and *ItfCDPK7* were induced significantly, whereas *ItfCDPK14* and *ItfCDPK18* were repressed by GA treatment. Under BAP treatment, *ItfCDPK11.2*, *-12.1*, *-12.2*, *-12.3*, *-9*, *-17.1*, *-33.1*, *-10*, *-30*, and *-18* were up-regulated (Figure 9A). In *I. triloba*, compared with Itb_Control, *ItbCDPK1*, *-11.2*, *-12.1*, *-12.3*, *-20.1*, *-9*, *-33.1*, *-33.2*, *-34*, *-10*, *-30*, and *-32* showed different expression patterns under hormone treatment compared with those in *I. trifida*. *ItbCDPK1* was up-regulated by ABA while *ItbCDPK11.2* was repressed by BAP. *ItbCDPK12.1* was induced by ABA and repressed by BAP. *ItbCDPK12.3* was up-regulated by GA. *ItbCDPK20.1* was induced by ABA. *ItbCDPK9* was induced by ABA but repressed by BAP. *ItbCDPK33.1* was induced by IAA but repressed by BAP. *ItbCDPK33.2* was induced by GA but not ABA. *ItbCDPK34* was not responsive to any hormone. *ItbCDPK10* was up-regulated by ABA but not BAP and down-regulated by IAA. *ItbCDPK30* was induced by ABA but not BAP. *ItbCDPK32* was induced but *ItfCDPK32* was repressed by ABA (Figure 9B). Furthermore, *IbCDPKs* and their homologous *CDPKs* in *I. trifida* and *I. triloba* showed different expression patterns in response to ABA, GA, IAA, and MeJA. These results indicated that *CDPKs* might function in developmental processes through various hormone signaling pathways between sweet potato and its two diploid relatives.

#### 2.6.3. Expression Analysis under Abiotic Stresses

**Figure 10:**
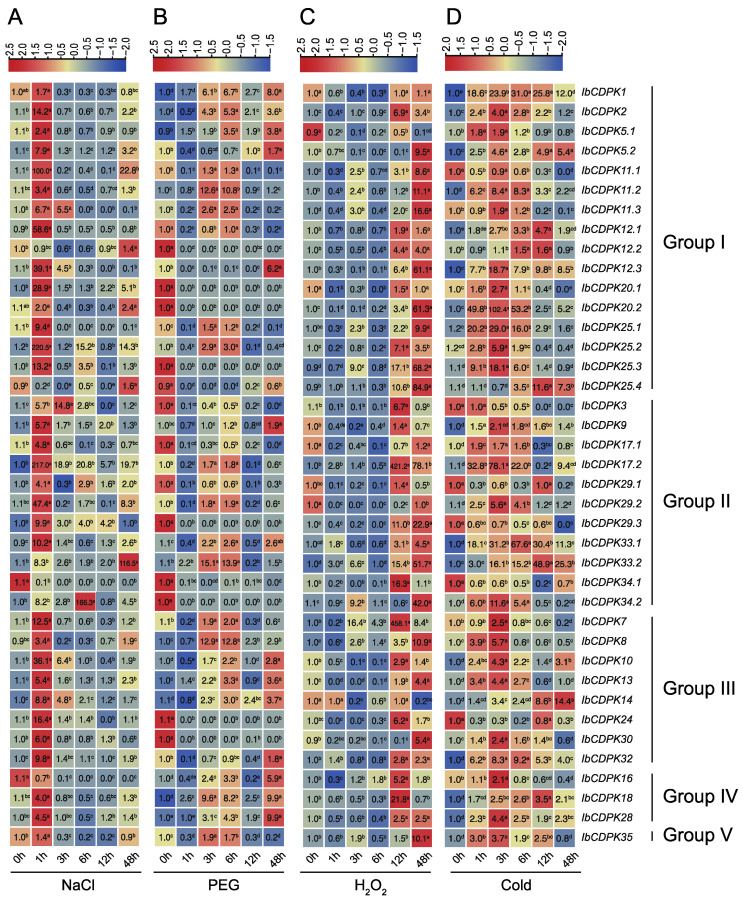
Gene expression patterns of *IbCDPKs* in response to abiotic stresses, i.e., (**A**) NaCl, (**B**) PEG, (**C**) H_2_O_2_, and (**D**) cold, of *I. batatas.* The values were determined by RT-qPCR from three biological replicates consisting of pools of three plants, and the results were analyzed using the comparative C_T_ method. The expression of 0 h in each treatment was considered “1”. The fold change is shown in the boxes. Different lowercase letters indicate significant differences (*p* < 0.05; Student’s *t*-test).

To evaluate the possible function of *IbCDPKs*, the level of transcript accumulation was determined using quantitative real-time PCR in leaf tissues at 0, 1, 3, 6, 12, and 48 h under NaCl, PEG, H_2_O_2_, and cold treatments (Figure 10). The result illustrated a variable transcript accumulation at different time points post stresses. Under salt stress, the majority of *IbCDPKs* were up-regulated with four genes, *IbCDPK11.1*, *IbCDPK17.2*, *IbCDPK33.2*, and *IbCDPK34.2*, induced by more than 100-fold. They peaked at 1 h except *IbCDPK12.2* (48 h), *IbCDPK33.2* (48 h), and *IbCDPK34.2* (6 h). Only three *IbCDPKs* (i.e., *IbCDPK25.4*, *IbCDPK34.1*, and *IbCDPK16*) were down-regulated (Figure 10A). Under drought stress, more than half of the *IbCDPKs* were up-regulated, but thirteen *IbCDPKs* (i.e., *IbCDPK12.2*, *-20.1 -20.2, -25.3, -25.4, -3, -17.1, -29.1, -29.3, -34.1, -34.2, -24*, and *-30*) were repressed (Figure 10B). Under oxidation stress, only five *IbCDPKs* (i.e., *IbCDPK1*, *-5.1*, *-17.1, -29.2*, and *-14*) were repressed. *IbCDPK17.2* and *IbCDPK7* were up-regulated by more than 400-fold. The expression levels of induced *IbCDPKs* peaked at 12 h or 48 h (Figure 10C). Under cold stress, thirty-three *IbCDPKs* were induced with *IbCDPK20.2* up-regulated by 102.4-fold, whereas six *IbCDPKs* (i.e., *IbCDPK11.1, -3, -29.1, -29.3, -34.1*, and *-24*) were significantly repressed (Figure 10D). In general, twenty *IbCDPKs* were induced by all four abiotic stress treatments in sweet potato, while only *IbCDPK34.1* was down-regulated in three abiotic stress treatments (NaCl, PEG, and cold). These results indicated *IbCDPKs* might be key players in abiotic stress resistance.

**Figure 11:**
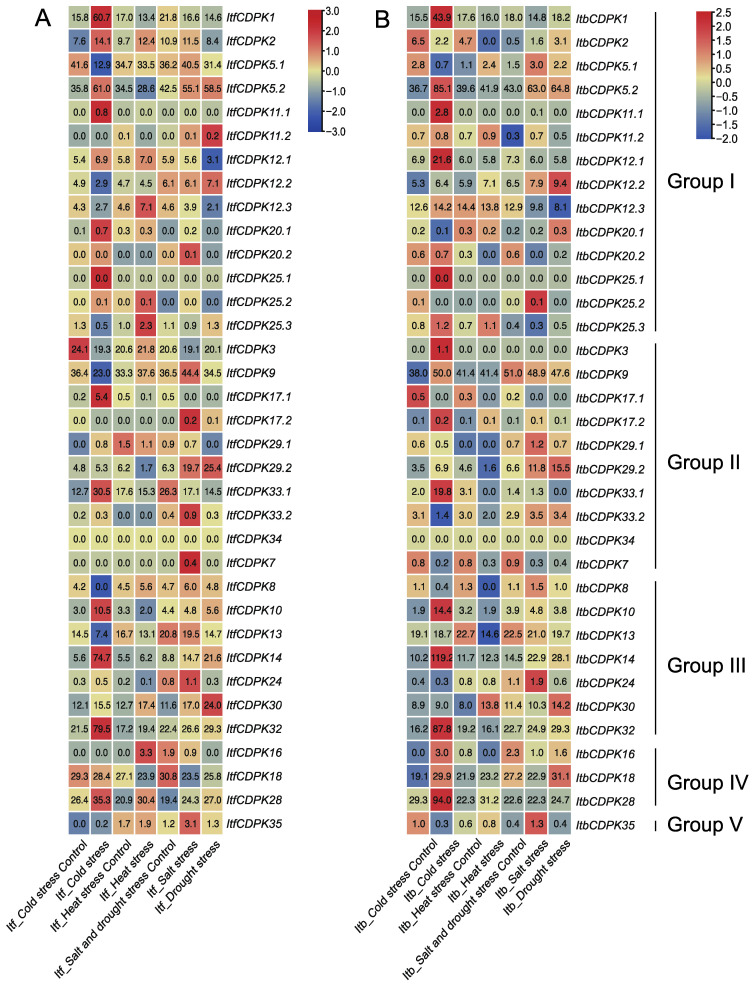
Gene expression patterns of *ItfCDPKs* (**A**) and *ItbCDPKs* (**B**) under abiotic stress (cold, heat, salt, and drought) in *I. trifida* and *I. triloba* as determined by RNA-seq. Log_2_ (FPKM+1) is shown in the boxes.

In addition, we also analyzed the expression patterns of *CDPKs* using the RNA-seq data of *I. trifida* and *I. triloba* under cold, heat, drought, and salt treatments [38]. In *I. trifida*, under cold treatment, compared with the control, in group I, *ItfCDPK1, -2, -5.2, -11.1, -12.1, -20.1, -20.2, -25.1, -25.2* were induced and *ItfCDPK5.1*, *-12.2*, *-12.3*, *-25.3* were repressed. In group II, *ItfCDPK*17.1 and *ItfCDPK*33.1 were induced and *ItfCDPK3, -9,* and *-17.2* were repressed. *ItfCDPK10, ItfCDPK14,* and *ItfCDPK32* were induced while *ItfCDPK8* and *ItfCDPK13* were repressed. *ItfCDPK28* was induced. The results suggested these *ItfCDPK*s might be involved in the response to cold stress. *ItfCDPK2*, *-12.1*, *-12.3*, *-25.2*, *-25.3* in group I, *-16* and *-28* in group IV were induced by heat treatment. *ItfCDPK20.2*, *-9*, *-17.2*, *-29.2*, *-33.2*, *-7*, *-24* and *-35* were induced by salt stress, significantly. Under drought stress, *ItfCDPK11.2*, *-12.2*, *-29.2*, and *-30* showed higher expression levels, while *ItfCDPK12.1*, *-12.3*, *-20.1*, *-20.2*, *-29.1*, *-33.1*, *-24*, *-18*, and *-28* showed lower expression levels. *ItfCDPK2* was induced by cold, heat, salt stresses. *ItfCDPK5.2* was induced by cold, salt, and drought stresses. *ItfCDPK12.1* and *ItfCDPK28* were induced by cold and heat stresses. *ItfCDPK29.2* was induced by salt and drought stresses. *ItfCDPK30* was induced by cold, heat, salt, and drought (Figure 11A). In *I. triloba*, *ItbCDPK2* showed an opposite expression pattern in cold, heat, salt, and drought stresses compared with *ItfCDPK2*. *ItbCDPK12.2* was induced by cold stress. *ItbCDPK20.1* was repressed by cold stress. *ItbCDPK20.2* was repressed in salt stress. *ItbCDPK3* and *ItbCDPK9* were induced by cold stress. *ItbCDPK17.1* and *ItbCDPK17.2* were repressed by cold stress. *ItbCDPK7* was repressed by salt stress. *ItbCDPK16* was induced by cold stress and repressed by heat stress. *ItbCDPK18* was induced by drought stress (Figure 11B). These results indicated that *CDPKs* showed commonalities and differences in response to abiotic stresses in *I. trifida* and *I. triloba.*

## 3. Discussion

CDPKs are essential in the regulation of plant growth and development, as well as in response to biotic and abiotic stresses. In the previous study, *CDPKs* were identified in *Arabidopsis*, rice, wheat, maize, polar, pear, and grape. However, the functional roles of the *CDPKs* family are still poorly understood in sweet potato. The modern cultivated sweet potato (*I. batatas*) is an autohexaploid (2n = 6x = 90) varying from *I. trifida* NCNSP0306 (2n = 2x = 30) and *I. triloba* NCNSP0323 (2n = 2x = 30) (Figure S1) and is an important crop because of its tuberous roots [38]. In this work, we characterized the *CDPKs* family in sweet potato and its two diploid relatives using whole-genome sequence data. To investigate physicochemical properties of *CDPKs*, the protein physiological properties, chromosome localization, phylogenetic relationships, conserved motifs, and protein interaction networks were predicted. We also analyzed the expression patterns of *CDPKs* in different tissues and relatives cross-talking of multiple stress signaling pathways. Genome-wide identification of *CDPKs* in sweet potato and its two diploid relatives will facilitate further genetic studies of growth, development, and stress resistance.

### 3.1. Evolution of the CDPK Gene Family in Sweet Potato and Its Two Diploid Relatives

A total of 109 *CDPKs* (i.e., 39 in *I. batatas*, 35 in *I. trifida*, and 35 in *I. triloba*) were identified from the cultivated hexaploid sweet potato and its two diploid relatives. According to the evolutionary distance to AtCPKs, these CDPK*s* were classified into five subgroups (group I to V), with one more group than CDPK*s* in other species (group I to IV) [3,9,10,11,12]. Ib/Itf/ItbCDPK35 in group V have no homologous protein in *Arabidopsis*, meaning group V in sweet potato is unique. We also observed that the number of *CDPKs* varied between sweet potato and its two diploid relatives. Thirty-nine *IbCDPKs* were distributed across the genome in *I. batatas* (Figure 1A). While the number of *CDPKs* identified in *I. trifida* was the same as that in *I. triloba* but was less than that in *I. batatas*., supporting the distinct origin during the evolution of hexaploid sweet potato.

In this study, a total of 10 motifs were identified in the 109 CDPKs from *I. batatas*, *I. trifida*, and *I. triloba* (Figure 3A and Figure S2), including six protein kinase domains and four EF-hands. Moreover, these motifs were highly conserved in *I. batatas*, *I. trifida*, and *I. triloba*. The majority of CDPKs contained a protein kinase domain and EF-hand except for ItbCDPK13, which contained a protein kinase domain only. The EF-hand in the C-terminus is a Ca^2+^ binding site [59], which means IbCDPK13 might be Ca^2+^ insensitive. There was no significant difference in each group except for Ib/Itf/ItbCDPK35 in group V. The number of protein kinase domains and EF-hands varied in sweet potato and its two diploid relatives, especially in group I (i.e., ItfCDPK5.1, Ib/ItbCDPK11.1, IbCDPK11.3, IbCDPK12.1, IbCDPK12.3, ItbCDPK20.1, Ib/ItbCDPK20.2, ItbCDPK25.1, Ib/Itf/ItbCDPK25.2, and IbCDPK25.4) (Figure 3A).

Besides, the exon-intron structures of some homologous *CDPKs* were different between sweet potato and its two diploid relatives and varied from *I. trifida* and *I. triloba* also. Some *CDPKs* contained the same number of exons and introns in *I. trifida* and *I. triloba* but less than that in *I. batata,* while some *CDPKs* contained different exons-introns in *I. batatas*, *I. trifida*, and *I. triloba*. These results suggested that *CDPKs* produced more changes during the evolution from diploid to hexaploid, while the structures of other groups were relatively conserved. Moreover, *CDPKs* in group I might play more critical roles in plant growth and development and response to environmental stress.

### 3.2. Different Functions of CDPKs on Growth and Development between Sweet Potato and Its Two Diploid Relatives

*CDPKs* could be detected on roots, stems, leaves, fruits, and seeds of plants. The expression levels of *OsCDPK2* increased with the seed development period. The expression patterns of *OsCDPK2* were opposite in green leaves exposed to light and darkness. *OsCDPK2* might function in seed development and in response to light in leaves [60]. *PiCDPK1* and *PiCDPK2* were specifically expressed at the pollen stage in *Petunia hybrida*. Overexpression of *PiCDPK1* disturbed the growth polarity of pollen tubes, while overexpression of *PiCDPK2* inhibits the elongation ability of pollen tubes but had no effect on the growth polarity of pollen tubes [52]. *PnCDPK1* was accumulated mainly in petals and sepals, which means that *PnCDPK1* may be an important component in the signal transduction pathways for flower morphogenesis [30]. Here, the expression levels of *CDPKs* in different tissues of *I. batatas*, *I. trifida*, and *I. triloba* were shown (Figure 6 and Figure 7). In *I.batatas*, no similar expression trends were observed between subgroups in five tissues. The majority of *IbCDPKs* were highly expressed in leaf, petiole, and pigmented root. Many *IbCDPKs* showed tissue-specific expression, and some *IbCDPKs* showed higher expression levels in the same tissue at the same time except for in tuberous root. Interestingly, only *IbCDPK28* showed the highest expression in tuberous root (Figure 6). *IbCDPK28* might be a key regulator in the development of tuberous root. In *I. trifida*, most *ItfCDPKs* in group I and group II might play key roles in flower and flowerbud growth and development. *ItfCDPKs* in group III might play roles in stem and flower and *ItfCDPKs* in group IV and V might be involved in root growth and development (Figure 7A). In *I. triloba*, the expression pattern *of ItbCDPKs* was similar to that *of ItfCDPKs* in *I. trifida* except for *ItbCDPK1*, *-2*, *-12.2*, and *-25.2* (Figure 7B). Some homologous *CDPKs* showed similar or opposite tissue-specific expression (Figure 6 and Figure 7). *Ib/Itf/ItbCDPK5.1* were highly expressed in stem and *Ib/Itf/ItbCDPK18* were highly expressed in leaf. *IbCDPK3* was lowly expressed, whereas *ItfCDPK3* was highly expressed in leaf. Diverse gene expression patterns among homologous *CDPKs* across different tissues suggest that CDPK proteins might be specialized for different biological responses.

### 3.3. Different Functions of CDPKs on Hormone Crosstalk between Sweet Potato and Its Two Diploid Relatives

Protein interaction prediction was performed to further reveal the potential function of IbCDPKs (Figure 5). IbCDPKs might interact with each other, such as IbCDPK11 and IbCDPK24, although it has not been reported that CDPKs could form homodimers. They might interact with JA biosynthesis-related proteins (i.e., ACX1 and ACX5) or ABA-responsive element-binding factor 1 (ABF1). IbCDPK11.1, -11.2, -12.1, -12.2, and -12.3 might interact with DI19 in response to drought stress. In rice, OsDi19-4 regulated the expression of *OsASPG1* and *OsNAC18*, two ABA-responsive genes, by directly binding to their promoters. The regulation was further enhanced by the increased phosphorylation of OsDi19-4 after the treatment of ABA [53]. Furthermore, All *IbCDPKs* were induced by at least two hormones (Figure 8). The majority of *IbCDPKs* possessed at least two hormone-responsive elements, such as ABA-responsive elements (ABRE, GARE-motif), MeJA-responsive elements (CGTCA-motif and TGACG-motif), GA-responsive elements (P-box and TATC-box), SA-responsive element (TCA), auxin-responsive elements (TGA-element) (Figure 4). Moreover, most of them peaked within 12 h (Figure 8), indicating that *IbCDPKs* could sense fluctuations quickly in hormone levels. SLAC1 was regulated by two AtCDPK protein kinases (CPK21 and CPK23), with distinct Ca^2+^ affinities in response to drought stress through ABA signaling pathways [54]. In *Arabidopsis*, disruption of the *CPK6* gene impaired MeJA-induced stomatal closure. MeJA-induced transient cytosolic free calcium concentration increments were reduced in the *cpk6-1* mutant. MeJA failed to activate slow-type anion channels in the *cpk6-1* guard cells. *AtCPK6* functions as a positive regulator of MeJA signaling in Arabidopsis guard cells [26]. Overexpression of *ZmCPK4* in the transgenic *Arabidopsis* enhanced ABA sensitivity in seed germination, seedling growth, and stomatal movement. The transgenic plants also enhanced drought stress tolerance [55]. Thus, *IbCDPKs* might also participate in hormone signaling pathways in response to environmental stress.

However, some of their homologous *CDPKs* in *I. trifida* and *I. triloba* showed different expression patterns in response to ABA, GA, IAA, and MeJA. Under IAA treatment, *ItfCDPKs* and *ItbCDPKs* were insensitive. For example, *Ib/Itf/ItbCDPK1*, *-5.1*, *-25.3*, *-16*, and *-35* under ABA treatment, *-12.3*, *-9*, and *-10* under IAA treatment, and *-18* under GA treatment showed opposite expression trends (Figure 8; Figure 11). In addition, expression patterns of *ItfCDPKs* were not exactly the same as those of *ItbCDPKs* (i.e., *Itf/ItbCDPK30*, *-32* under ABA treatment, -*33.1* under IAA treatment, *-2* under GA treatment, and *-30* under BAP treatment) (Figure 11). These results indicated that *CDPKs* participated in multiple hormones crosstalk, and homologous *CDPK* genes were involved in different hormonal pathways in sweet potato and its two diploid relatives.

### 3.4. Different Functions of CDPKs on Multiple Abiotic Stress Response between Sweet Potato and Its Two Diploid Relatives

There have been many reports that *CDPKs* were related to abiotic stress resistance [32,33,34]. In *Chenopodium glaucum*, *CgCDPK* interacted with *CgbHLH001* in the signal transduction pathway in response to salt and drought stress [56]. Overexpression of *SiCDPK24* in *Arabidopsis* enhanced drought resistance and improved the survival rate under drought stress [61]. In this study, we analyzed the level of transcript accumulation using qRT-PCR at different time points post treatments (Figure 10). Under NaCl, H_2_O_2_, and cold stresses, the expression of *IbCDPKs* peaked at 1 h (Figure 10A), 12/48 h (Figure 10C), and 3 h (Figure 10D), meaning that the regulation of *IbCDPKs* was mainly activated on the prophase of NaCl and cold treatments, and the anaphase of H_2_O_2_ treatment, respectively. In general, thirty-eight *IbCDPKs* were up-regulated by at least two abiotic stresses, consistent with *CDPK* genes in other species. The majority of *IbCDPKs* were induced by NaCl, H_2_O_2_, and cold stresses. *IbCDPK25.3* was up-regulated 12.2-fold at 1 h under NaCl stress, 68.2-fold at 48 h under H_2_O_2_, and 18.1-fold at 3 h under cold stress, respectively. *IbCDPK33.2* was induced under four stresses, with 116.5-fold at 48 h under NaCl stress, 15.1-fold at 3 h under PEG stress, 51.7-fold at 48 h under H_2_O_2_ stress, and 48.9-fold at 12 h under cold stress, respectively (Figure 9). In protein interaction prediction, IbCDPKs might interact with a potassium channel protein (KAT2), stomatal movement protein (ELUS3) (Figure 5). These results indicated that IbCDPKs might be key players in response to abiotic stresses by regulating stomatal movement and icon transport.

In addition, the expression patterns of homologous *CDPKs* in diploid *I. trifida* and *I. triloba* using RNA-seq were distinct (Figure 11). The numbers of *Itf/ItbCDPKs* induced by salt and cold stresses were less than those of *IbCDPKs*, which may be due to the fact that only one time point was detected.

Differences in expression patterns of *CDPKs* in sweet potato and its two diploid relatives might provide potential candidate genes for further functional characterization and for improving abiotic stress tolerance of sweet potato and other species.

## 4. Materials and Methods

### 4.1. Identification of CDPKs

The whole-genome sequences of *I. batatas*, *I. trifida*, and *I. triloba* were downloaded from *Ipomoea* Genome Hub (https://ipomoea-genome.org/, accessed on 15 January 2022) and Sweetpotato Genomics Resource (http://sweetpotato.plantbiology.msu.edu/, accessed on 15 January 2022). To accurately identify all *CDPKs* family members, two different screening methods were combined. Firstly, the HMMER 3.0 software was used to identify potential *CDPKs* through the Hidden Markov Model profiles (hmmsearch, E value ≤ 1 × 10^−5^) of Pkinase domain (PF00069) and EF-hand 7 (PF13499), which were extracted from the Pfam databases (http://pfam.xfam.org/ accessed on 30 November 2021). Next, all putative CDPKs were retested using SMART (http://smart.embl-heidelberg.de/ accessed on 1 December 2021) and CD-search (https://www.ncbi.nlm.nih.gov/Structure/cdd/wrpsb.cgi accessed on 1 December 2021).

### 4.2. Chromosomal Distribution of CDPKs

The *IbCDPKs*, *ItfCDPKs*, and *ItbCDPKs* were separately mapped to the *I. batatas*, *I. trifida*, and *I. triloba* chromosome based on the chromosomal location provided in the *Ipomoea* Genome Hub (https://ipomoea-genome.org/ accessed on 15 January 2022) and Sweetpotato Genomics Resource (http://sweetpotato.plantbiology.msu.edu/ accessed on 15 January 2022). The visualization was generated by the TBtools software (v.1.098696) (South China Agricultural University, Guangzhou, China) [62].

### 4.3. Protein Properties Prediction of CDPKs

The MW, pI, and the number of EF-hands of CDPKs were calculated by ExPASy (https://www.expasy.org/ accessed on 23 December 2021). The N-myristoylation and Palmitoylation sites of CDPKs were predicted by GPS-Lipid 1.0 with a high threshold (http://lipid.biocuckoo.org/ accessed on 24 December 2021) [63].

### 4.4. Phylogenetic Analysis of CDPKs

The phylogenetic analysis of CDPKs from *I. batatas*, *I. trifida*, *I. triloba*, and *Arabidopsis* was performed using ClustalW in MEGA X [64] with default parameters, the maximum likelihood method, and the Poisson correction model. Bootstrapping was performed with 1000 replicates. Then, the phylogenetic tree was constructed by iTOL (http://itol.embl.de/ accessed on 13 January 2022).

### 4.5. Domain Identification and Conserved Motifs Analysis of CDPKs

The conserved motifs of CDPKs were analyzed by MEME (https://meme-suite.org/meme/ accessed on 29 January 2022), the maximum number of motifs parameter was set to 10.

### 4.6. Exon–Intron Structures and Promoter Analysis of CDPKs

The exon-intron structures of *CDPKs* were obtained by GSDS 2.0 (http://gsds.gao-lab.org/ accessed on 29 January 2022) and were visualized by the TBtools software. The *cis*-elements in the approximately 2000 bp promoter region of *CDPKs* were predicted by PlantCARE (http://bioinformatics.psb.ugent.be/webtools/plantcare/html/ accessed on 30 January 2022) [65].

### 4.7. Protein Interaction Network of CDPKs

The protein interaction network of CDPKs was predicted by STRING (https://cn.string-db.org/, accessed on 25 January 2022) based on *Arabidopsis* homologous proteins. The network map was built by Adobe Illustrator CC2019 software (Adobe Systems Incorporated, San Jose, CA, USA).

### 4.8. qRT-PCR Analysis of CDPKs

The salt-tolerant sweet potato (*I. batatas*) line ‘ND98’ was used for qRT-PCR analysis in this study [45]. In vitro-grown ND98 plants were cultured on Murashige and Skoog (MS) medium at 27 ± 1 °C under a photoperiod consisting of 13 h of cool-white fluorescent light at 54 μmol m^–2^ s^–1^ and 11 h of darkness. Sweet potato plants were cultivated in a field at the campus of China Agricultural University, Beijing, China.

For expression analysis in various tissues, total RNA was extracted from the pigmented root, tuberous root, stems, leaves, and petioles tissues of 3-month-old field-grown ND98 plants using the TRIzol method (Invitrogen). For expression analysis of hormone and abiotic treatment, the leaves were sampled at 0, 1, 3, 6, 12, and 48 h after being treated with 200 mM NaCl, 20% polyethylene glycol (PEG) 6000, 10 mM H_2_O_2_, 4 °C, 100 μM ABA, 100 μM GA, 100 μM MeJA, and 100 μM IAA, respectively. Three independent biological replicates were taken, each with three plants. qRT-PCR was conducted using the SYBR detection protocol (TaKaRa, Kyoto, Japan) on a 7500 Real-Time PCR system (Applied Biosystems, Foster City, CA, USA). The reaction mixture was composed of first-strand cDNA, primer mix, and SYBR Green M Mix (TaKaRa; code RR420A) to a final volume of 20 μL. A sweet potato actin gene (GenBank AY905538) was used as an internal control. The relative gene expression levels were quantified with the comparative C_T_ method [66]. The specific primers of qRT-PCR analysis are listed in Table S2. The heat maps of gene expression profiles were constructed using TBtools software (v.1.098696) [62].

### 4.9. Transcriptome Analysis

The RNA-seq data of *ItfCDPKs* and *ItbCDPKs* in *I. trifida* and *I. triloba* were downloaded from the Sweetpotato Genomics Resource (http://sweetpotato.plantbiology.msu.edu/ accessed on 20 January 2022). The expression levels of CDPKs were calculated as fragments per kilobase of exon per million fragments mapped (FPKM). The heat maps were constructed by TBtools software (v.1.098696) [62].

## 5. Conclusions

Here, we identified 39, 35, and 35 *CDPKs* in cultivated hexaploid sweet potato and its two diploid relatives, *I. trifida* and *I. triloba,* respectively. There were differences in chromosome localization, phylogenetic relationship, and gene structure of these 109 *CDPKs*. The expression profiles of the identified *CDPKs* indicated that *CDPKs* showed tissue specificity and various expression patterns in sweet potato and its two diploid relatives. These results indicated that homologous *CDPKs* might be involved in distinct hormone crosstalk and abiotic stress responses to regulate plant growth and development. Moreover, the identification of interacting proteins of each CDPK might be their phosphorylation targets to help identify the mechanism. This work provided valuable insights into the structure and function of *CDPK* genes and provided more potential candidate genes for improving field and abiotic stress tolerance in sweet potato and its two diploid relatives.

## Figures and Tables

**Table 1:** Characterization of *IbCDPKs* in sweet potato.

Gene Name	Gene ID	CDS Length/bp	Protein Size/aa	MW/kDa	pI	No. of EF-Hands	N-Myristoylation	Palmitoylation
*IbCDPK1*	Ib01g3472	1755	584	65.253	5.72	4	No	No
*IbCDPK2*	Ib07g25704	1962	653	72.939	6	4	Yes	Yes
*IbCDPK3*	Ib05g19617	2058	685	77.012	5.55	4	Yes	Yes
*IbCDPK5.1*	Ib10g41295	1701	566	62.970	5.2	4	Yes	Yes
*IbCDPK5.2*	Ib14g59849	1689	562	63.084	5.49	4	Yes	Yes
*IbCDPK7*	Ib12g50922	1677	558	62.852	8.69	4	Yes	Yes
*IbCDPK8*	Ib03g11409	1602	533	59.869	6.15	4	Yes	Yes
*IbCDPK9*	Ib09g34428	1563	520	58.469	5.98	4	Yes	Yes
*IbCDPK10*	Ib13g53626	1770	589	66.286	7.16	4	Yes	Yes
*IbCDPK11.1*	Ib01g3989	1416	471	52.521	6.05	4	Yes	No
*IbCDPK11.2*	Ib02g8624	1632	543	60.666	5.31	4	No	Yes
*IbCDPK11.3*	Ib02g6000	1509	502	55.721	5.67	4	No	Yes
*IbCDPK12.1*	Ib07g27783	1530	509	57.151	5.01	4	No	No
*IbCDPK12.2*	Ib05g20844	1518	505	56.550	4.97	4	No	Yes
*IbCDPK12.3*	Ib09g35646	1668	555	62.731	5.71	3	No	No
*IbCDPK13*	Ib14g58861	1461	486	54.436	5.63	4	Yes	Yes
*IbCDPK14*	Ib14g58509	1614	537	60.600	6.25	4	Yes	Yes
*IbCDPK16*	Ib07g29399	3513	1170	130.824	8.09	4	Yes	Yes
*IbCDPK17.1*	Ib02g4428	1716	571	63.410	5.53	4	Yes	Yes
*IbCDPK17.2*	Ib12g50948	1575	524	58.850	5.31	4	Yes	Yes
*IbCDPK18*	Ib01g414	1866	621	70.113	8.65	3	Yes	Yes
*IbCDPK20.1*	Ib14g58329	1635	544	59.975	5.53	4	Yes	Yes
*IbCDPK20.2*	Ib06g25035	1548	515	56.855	5.02	4	Yes	Yes
*IbCDPK24*	Ib09g36010	1353	450	51.744	6.05	3	No	No
*IbCDPK25.1*	Ib06g25043	1911	636	71.191	5.89	4	No	No
*IbCDPK25.2*	Ib11g45599	1866	621	70.036	5.13	4	Yes	Yes
*IbCDPK25.3*	Ib14g58318	1767	588	65.341	5.56	4	Yes	Yes
*IbCDPK25.4*	Ib14g58307	1056	351	38.732	4.75	3	Yes	Yes
*IbCDPK28*	Ib05g16972	1800	599	67.199	9.28	4	Yes	Yes
*IbCDPK29.1*	Ib10g38551	1497	498	56.102	5.74	4	No	No
*IbCDPK29.2*	Ib15g60594	1443	480	53.887	4.87	4	Yes	Yes
*IbCDPK29.3*	Ib15g60591	1518	505	56.806	5.14	3	Yes	Yes
*IbCDPK30*	Ib08g33870	1692	563	63.786	6.08	4	Yes	Yes
*IbCDPK32*	Ib06g24869	1560	519	58.264	7.62	3	Yes	Yes
*IbCDPK33.1*	Ib01g217	1509	502	71.191	6.54	4	Yes	No
*IbCDPK33.2*	Ib15g61179	1554	517	57.706	5.88	4	Yes	Yes
*IbCDPK34.1*	Ib03g11293	1683	560	62.193	5.84	4	Yes	Yes
*IbCDPK34.2*	Ib03g11291	1578	525	58.304	5.49	4	Yes	Yes
*IbCDPK35*	Ib06g24207	1488	495	54.747	5.23	3	No	Yes

CDS, coding sequence; MW, molecular weight; pI, isoelectric point.

## Data Availability

The data presented in this study are available on request from the corresponding author.

## Supplementary Materials

The following are available online at: https://www.mdpi.com/article/10.3390/ijms23063088/s1.
