# Supplementary material for: Genome-Wide Identification and Characterization of CDPK Family Reveal Their Involvements in Growth and Development and Abiotic Stress in Sweet Potato and Its Two Diploid Relatives
Source: Int J Mol Sci. 2022 Mar 13;23(6):3088. doi: 10.3390/ijms23063088 (PMC8952862; doi:10.3390/ijms23063088)
Supplement: Supplementary file 1 [file ijms-23-03088-s001.zip › ijms-1614575-supplementary.pdf]

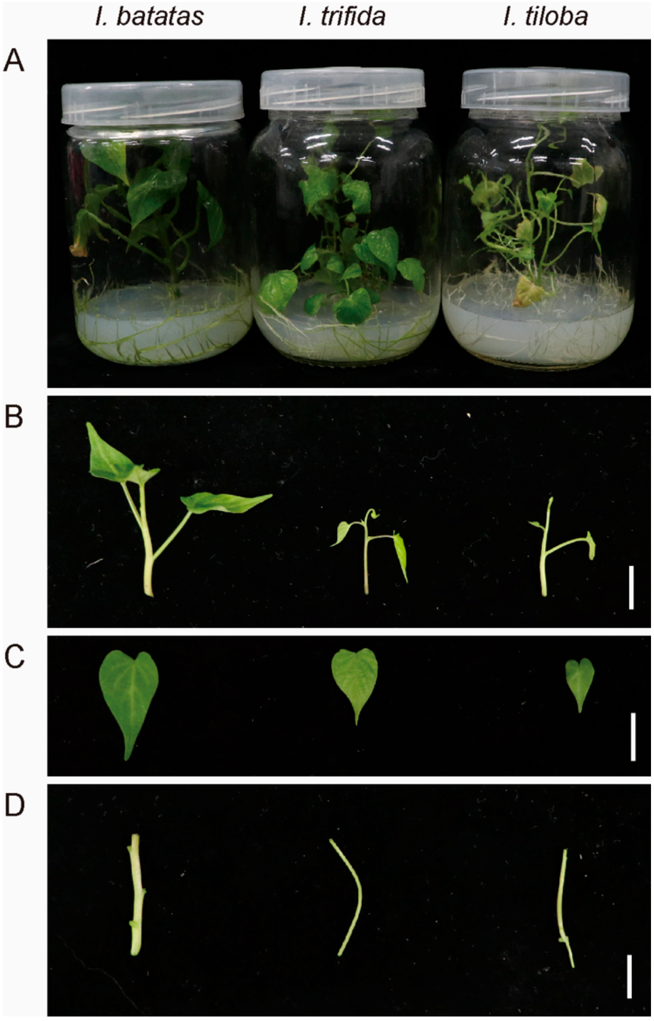

Figure S1. Morphology of *Ipomoea batatas*, *I. trifida*, and *I. triloba.* (A) *In vitro*-grown plants, (B) buds, (C) leaves and (D) stems of *I. batatas* "ND98", *I. trifida* NCNSP0306, and *I. triloba* NCNSP0323. Scale bars, 1cm.

Table S1. Identification of *CDPK* family genes in *I. batatas*, *I. trifida*, and *I. triloba*.

Arabidopsis	**Homologous gene in *I.batatas/I. trifida/ I. triloba***	**Gene ID**	**Gene name**	**Chromosome localization**
Group Ⅰ: AT5G04870/*CDPK1* AT3G10660/*CDPK2* AT4G09570/*CDPK4* AT4G35310/*CDPK5* AT2G17290/*CDPK6* AT1G35670/*CDPK11* AT5G23580/*CDPK12* AT2G38910/*CDPK20* AT2G35890/*CDPK25* AT4G38230/*CDPK26*	*I. batatas*	Ib01g3472	*IbCDPK1*	LG1:24830785-24835345
		Ib07g25704	*IbCDPK2*	LG7:2699303-2705437
		Ib10g41295	*IbCDPK5.1*	LG10:23284891-23292007
		Ib14g59849	*IbCDPK5.2*	LG14:30745372-30750537
		Ib01g3989	*IbCDPK11.1*	LG1:28751452-28754855
		Ib02g8624	*IbCDPK11.2*	LG2:32588144-32592507
		Ib02g6000	*IbCDPK11.3*	LG2:12279066-12283322
		Ib07g27783	*IbCDPK12.1*	LG7:18696882-18700991
		Ib05g20844	*IbCDPK12.2*	LG5:30407064-30411224
		Ib09g35646	*IbCDPK12.3*	LG9:11473256-11478686
		Ib14g58329	*IbCDPK20.1*	LG14:21473796-21478088
		Ib06g25035	*IbCDPK20.2*	LG6:30232923-30236999
		Ib06g25043	*IbCDPK25.1*	LG6:30276514-30279598
		Ib11g45599	*IbCDPK25.2*	LG11:30664898-30669882
		Ib14g58318	*IbCDPK25.3*	LG14:21402724-21405804
		Ib14g58307	*IbCDPK25.4*	LG14:21337074-21340153
	I. trifida	Itf05g05320.t1	*ItfCDPK1*	Chr05:5000266-5004899
		Itf03g16550.t1	*ItfCDPK2*	Chr03:13311929-13316225
		Itf08g15310.t1	*ItfCDPK5.1*	Chr08:14928027-14934826
		Itf09g00070.t1	*ItfCDPK5.2*	Chr09:37207-42492
		Itf05g01790.t1	*ItfCDPK11.1*	Chr05:1418963-1422348
		Itf04g21130.t1	*ItfCDPK11.2*	Chr04:22531420-22535675
		Itf03g27590.t1	*ItfCDPK12.1*	Chr03:23958615-23962697
		Itf12g27210.t1	*ItfCDPK12.2*	Chr12:23765779-23769672
		Itf10g12490.t1	*ItfCDPK12.3*	Chr10:15034446-15039526
		Itf09g12710.t1	*ItfCDPK20.1*	Chr09:7567316-7571332
		Itf15g02240.t1	*ItfCDPK20.2*	Chr15:1278659-1281946
		Itf15g02150.t1	*ItfCDPK25.1*	Chr15:1230203-1233049
		Itf01g08260.t1	*ItfCDPK25.2*	Chr01:6871006-6875288
		Itf09g12810.t1	*ItfCDPK25.3*	Chr09:7632433-7635670
	I. triloba	Itb05g04690.t1	*ItbCDPK1*	Chr05:4310327-4314941
		Itb03g17340.t1	*ItbCDPK2*	Chr03:15981183-15985409
		Itb08g16550.t1	*ItbCDPK5.1*	Chr08:18612128-18618594
		Itb09g00070.t1	*ItbCDPK5.2*	Chr09:170227-175626
		Itb05g01310.t1	*ItbCDPK11.1*	Chr05:1115236-1118389
		Itb04g20500.t1	*ItbCDPK11.2*	Chr04:25266912-25271378
		Itb03g26380.t2	*ItbCDPK12.1*	Chr03:25926382-25930586
		Itb12g27640.t1	*ItbCDPK12.2*	Chr12:27917080-27921089
		Itb10g13020.t1	*ItbCDPK12.3*	Chr10:19104450-19110550

		Itb09g13740.t1	*ItbCDPK20.1*	Chr09:8992317-8995794
		Itb15g02380.t1	*ItbCDPK20.2*	Chr15:1441612-1451578
		Itb15g02300.t1	*ItbCDPK25.1*	Chr15:1394597-1397355
		Itb01g12560.t1	*ItbCDPK25.2*	Chr01:12112283-12117224
		Itb09g13830.t1	*ItbCDPK25.3*	Chr09:9067762-9071145
Group Ⅱ：<br>AT4G23650/*CDPK3*<br>AT3G20410/*CDPK9*<br>AT4G21940/*CDPK15*<br>AT5G12180/*CDPK17*<br>AT1G61950/*CDPK19*<br>AT4G04720/*CDPK21*<br>AT4G04710/*CDPK22*<br>AT4G04740/*CDPK23*<br>AT4G04700/*CDPK27*<br>AT1G76040/*CDPK29*<br>AT4G04695/*CDPK31*<br>AT1G50700/*CDPK33*<br>AT5G19360/*CDPK34*	*I.batatas*	Ib05g19617	*IbCDPK3*	LG5:22129803-22135374
		Ib09g34428	*IbCDPK9*	LG9:2567297-2572974
		Ib02g4428	*IbCDPK17.1*	LG2:1691652-1694865
		Ib12g50948	*IbCDPK17.2*	LG12:30175165-30179535
		Ib10g38551	*IbCDPK29.1*	LG10:2597602-2602106
		Ib15g60594	*IbCDPK29.2*	LG15:4439385-4443335
		Ib15g60591	*IbCDPK29.3*	LG15:4418925-4422944
		Ib01g217	*IbCDPK33.1*	LG1:1168963-1172868
		Ib15g61179	*IbCDPK33.2*	LG15:8410101-8413448
		Ib03g11293	*IbCDPK34.1*	LG3:13809400-13813225
		Ib03g11291	*IbCDPK34.2*	LG3:13789742-13795902
	I. trifida	Itf12g18690.t1	*ItfCDPK3*	Chr12:18034787-18043398
		Itf10g21110.t1	*ItfCDPK9*	Chr10:22415094-22420328
		Itf04g33180.t1	*ItfCDPK17.1*	Chr04:31547514-31550480
		Itf07g01450.t1	*ItfCDPK17.2*	Chr07:796713-801278
		Itf08g02950.t1	*ItfCDPK29.1*	Chr08:2047133-2051513
		Itf06g21180.t1	*ItfCDPK29.2*	Chr06:22697131-22701278
		Itf05g25980.t1	*ItfCDPK33.1*	Chr05:24927675-24930598
		Itf06g16440.t1	*ItfCDPK33.2*	Chr06:19418232-19421675
		Itf14g09720.t2	*ItfCDPK34*	Chr14:8435021-8438271
	I. triloba	Itb12g18970.t2	*ItbCDPK3*	Chr12:21278101-21282504
		Itb10g21210.t1	*ItbCDPK9*	Chr10:26513343-26518575
		Itb04g32820.t1	*ItbCDPK17.1*	Chr04:35228752-35233296
		Itb07g01520.t1	*ItbCDPK17.2*	Chr07:919387-924182
		Itb08g03030.t1	*ItbCDPK29.1*	Chr08:2552604-2557130
		Itb06g19660.t1	*ItbCDPK29.2*	Chr06:22888417-22892731
		Itb05g26680.t1	*ItbCDPK33.1*	Chr05:30423254-30427294
		Itb06g14790.t1	*ItbCDPK33.2*	Chr06:19254127-19257539
		Itb14g13930.t1	*ItbCDPK34*	Chr14:16308224-16312921
Group Ⅲ：<br>AT5G12480/CDPK7<br>AT5G19450/CDPK8<br>AT1G18890/CDPK10<br>AT3G51850/CDPK13<br>AT2G41860/CDPK14<br>AT2G31500/CDPK24<br>AT1G74740/CDPK30<br>AT3G57530/CDPK32	*I.batatas*	Ib12g50922	*IbCDPK7*	LG12:30012193-30017201
		Ib03g11409	*IbCDPK8*	LG3:14627004-14632258
		Ib13g53626	*IbCDPK10*	LG13:18270694-18279128
		Ib14g58861	*IbCDPK13*	LG14:25089809-25095153
		Ib14g58509	*IbCDPK14*	LG14:22811668-22815893
		Ib09g36010	*IbCDPK24*	LG9:14310550-14314074
		Ib08g33870	*IbCDPK30*	LG8:26191741-26195526
		Ib06g24869	*IbCDPK32*	LG6:29208378-29212137
	I. trifida	Itf07g01710.t1	*ItfCDPK7*	Chr07:954406-959418
		Itf14g09010.t3	*ItfCDPK8*	Chr14:7627304-7632439

		Itf02g04140.t1	*ItfCDPK10*	Chr02:5200050-5205219
		Itf09g09360.t1	*ItfCDPK13*	Chr09:5200617-5205831
		Itf09g11960.t1	*ItfCDPK14*	Chr09:6957376-6961133
		Itf10g25830.t1	*ItfCDPK24*	Chr10:24769040-24772676
		Itf11g21340.t1	*ItfCDPK30*	Chr11:18573725-18577623
		Itf15g03330.t1	*ItfCDPK32*	Chr15:1971367-1975425
	I. triloba	Itb07g01790.t1	*ItbCDPK7*	Chr07:1087642-1092508
		Itb14g13060.t1	*ItbCDPK8*	Chr14:14981121-14986426
		Itb02g12420.t1	*ItbCDPK10*	Chr02:8485298-8490453
		Itb09g10160.t1	*ItbCDPK13*	Chr09:6317662-6323291
		Itb09g13020.t1	*ItbCDPK14*	Chr09:8333129-8337006
		Itb10g11150.t1	*ItbCDPK24*	Chr10:16611886-16615966
		Itb11g23150.t1	*ItbCDPK30*	Chr11:25070971-25074860
		Itb15g03590.t1	*ItbCDPK32*	Chr15:2273843-2278005
Group Ⅳ：AT2G17890/*CDPK16* AT4G36070/*CDPK18* AT5G66210/*CDPK28*	*I.batatas*	Ib07g29399	*IbCDPK16*	LG7:30194492-30205186
		Ib01g414	*IbCDPK18*	LG1:2258185-2262845
		Ib05g16972	*IbCDPK28*	LG5:2210945-2216258
	I. trifida	Itf03g06870.t1	*ItfCDPK16*	Chr03:4401137-4406030
		Itf05g23880.t1	*ItfCDPK18*	Chr05:23880059-23885122
		Itf12g02710.t1	*ItfCDPK28*	Chr12:1632109-1638134
	I. triloba	Itb03g06930.t1	*ItbCDPK16*	Chr03:5010301-5015125
		Itb05g24590.t1	*ItbCDPK18*	Chr05:29316001-29321092
		Itb12g02840.t1	*ItbCDPK28*	Chr12:1862753-1869785
Group V	*I.batatas*	Ib05g19617	*IbCDPK3*	LG5:22129803-22135374
	I. trifida	Itf15g08760.t1	*ItfCDPK35*	Chr15:5743579-5746107
	I. triloba	Itb15g09200.t1	*ItbCDPK35*	Chr15:6566782-6569097

Table S2. Primers used in this study.

Gene	Forward Primer	Reverse Primer
IbCDPK1	GCCTATGGCGGTTCAAAGTA	TCATCCGTCTCCGTTTTGG
IbCDPK2	CCAAAATAAGCCGCCTGAG	AACACCGAATCAGTCCTAAGTCC
IbCDPK3	TCTGTTGGGCAGCAGGTTT	CCCAACTCGGTGTTCTCCTC
IbCDPK5.1	TCATTCCATCTCCAAGCGTAA	TGTTCTGGGAAGAGGTCAAGC
IbCDPK5.2	TTCCATTTCCAACTGTAACCCT	GGTTGGGCTGGGTATCCTT
IbCDPK7	TCTTCTGGGAACAGGGCTAAG	GGTTGGGCTTCTTCTTGTCC
IbCDPK8	GGGCCAAGAATGAGAATAGAAA	GCTGTGAATGCTATCCCCTGTA
IbCDPK9	TAGGCAGGGAAGAGGAGTGG	GAGGATGGGGCTGTGGATTA
IbCDPK10	GCAAAAGCCGATTACAAGCC	GATTCGGGCTTTGGGGAT
IbCDPK11.1	ATGACAGGAGGGAAAGCAAAG	GTCCCTCAGCCTTGGTGTTT
IbCDPK11.2	CGCTTACCACTACGCCAAAA	CAGCACCCTGACTCTGGAAA
IbCDPK11.3	AGACCTTTGGGTTCAGCAGTT	GATTTCTTCTTTTCAGACCTCCC
IbCDPK12.1	GGGTGTCGGAAGAAGAACCA	TCCTCCCCATGCTTCTCATC
IbCDPK12.2	AAGGCAATGGAGGCGGAG	CATTATTGTCTACAAGTCCTAGCGC
IbCDPK12.3	ACTTTCCACCGACTTCCTTTG	CGTGAAAGAACCGCAGAGTC
IbCDPK13	TAGGGAGGATGTGAAGTCGGG	TCCTTCTTCACCTCAGTCAGCA
IbCDPK14	GCAAAATGCTCAGACGGCT	AGAGCTCTTTTCTTCAGCTTGTTC
IbCDPK16	ACCGCAAGACGACCACGAA	CCTGGCTTTGTTGGACGCT
IbCDPK17.1	AAGGAGGACGGAGAAGCACC	CAACCTGCGATGACCCTGAG
IbCDPK17.2	CAATCGGACCAAAATCAGGA	CCCTAGCACCGTCCCTATCT
IbCDPK18	TGAGACCACTGCCACATCAAC	CCCACAAGGGATAACCACAGT
IbCDPK20.1	TTTCTGCGGCGGTTTGG	GCCACTTTCTTGATATGCGACT
IbCDPK20.2	GACGGAGATTCCAATAATGAGC	TATGTTCCCTGTCTTCCTCCC
IbCDPK24	GCAATGTGACTCGGGAGATG	TTGTCCGTCCGTACCGATC
IbCDPK25.1	CCTGTTTTGGTGGTTTCGG	GGGGCTTATTGGTCTGAACTG
IbCDPK25.2	AGAGGTGGTGAGGATGGAGG	CATCCCGTTTTGTGGTCTCA
IbCDPK25.3	CTCAGAGGCTAAAGAGGCAGAA	TTTCCTTGTCATTTCCTCCTGT
IbCDPK25.4	ATGGGATCTTTCATAAACTCTTTTG	GTCCTTCAAATTCCCGGTTT
IbCDPK28	TGTTGCCCCAGAGGTATTGA	GCCGACCACAGAGCAAAATA
IbCDPK29.1	CTCAAGGAAGATGGCAATGC	GACCTTCAGAGCAAGCTGCTT
IbCDPK29.2	TGGGAAAGCCGTATGTGGA	GCAGCATCAAAACCTCCCTC
IbCDPK29.3	TCGCCCAGTCCCCATTGTA	CCACATACGGCTTCCCAAGA
IbCDPK30	GAATCCGCCGCCAAGAA	TTCGGGTTTTGGGGATGA
IbCDPK32	CAAGCCAAACCCGTTCTCC	GTGGGGTTTTCCAGCACATA
IbCDPK33.1	GGAAGCCATCAGACTCACATTG	GATTTTGGGATTGATTGTGGC
IbCDPK33.2	ATGGGTATCATGGTGGAATGG	CTGGTTTTGGGCTTGGTTTAG
IbCDPK34.1	CAAGGAGGATGGAGAAGCACC	GCACCCTGCTATCACCCTGA
IbCDPK34.2	ATGGGTCATCCGCAGCAA	TGCCCTATCTTCCCACCTGA
IbCDPK35	TAGCGAAACAGGATGAGATGGA	CAAGGAGAAGCTGCTGCTGAG
Ibactin	AGCAGCATGAAGATTAAGGTTGTAGCAC	TGGAAAATTAGAAGCACTTCCTGTGAAC

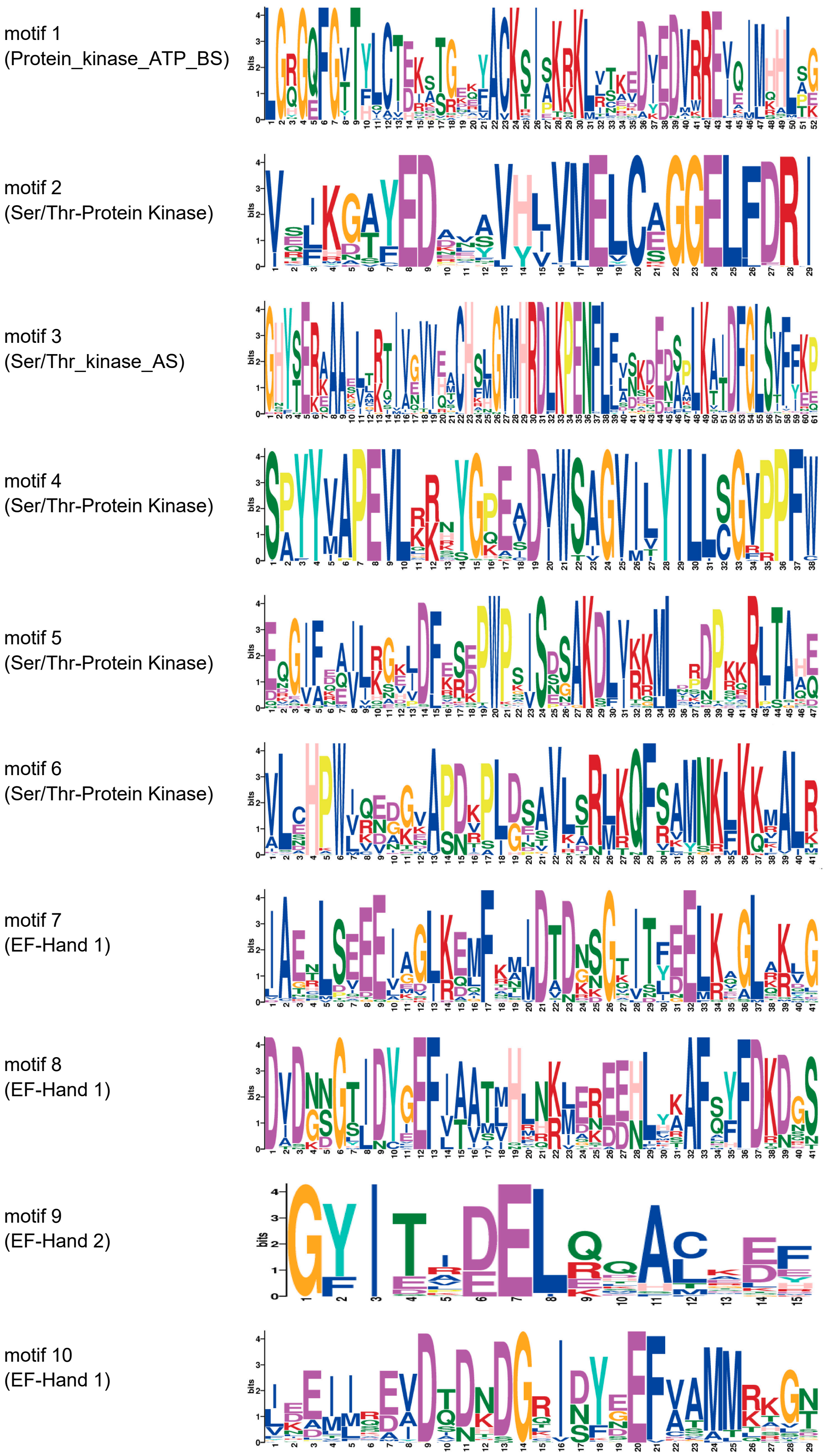

Figure S2. Conserved motifs analysis of IbCDPKs in *I. batatas*.
